# Identification and Expression Analysis of Calcium-Dependent Protein Kinases Gene Family in Potato Under Drought Stress

**DOI:** 10.3389/fgene.2022.874397

**Published:** 2022-05-24

**Authors:** Simon Dontoro Dekomah, Yihao Wang, Tianyuan Qin, Derong Xu, Chao Sun, Panfeng Yao, Yuhui Liu, Zhenzhen Bi, Jiangping Bai

**Affiliations:** ^1^ College of Agronomy, Gansu Agricultural University, Lanzhou, China; ^2^ Gansu Provincial Key Laboratory of Aridland Crop Science, Lanzhou, China

**Keywords:** calcium-dependent protein kinases, expression profiles, protein sequences, genes, drought conditions, cultivar

## Abstract

Calcium-dependent protein kinases (CDPKs) are a class of serine/threonine protein kinases encoded by several gene families that play key roles in stress response and plant growth and development. In this study, the BLAST method was used to search for protein sequences of the potato Calcium-dependent protein kinase gene family. The chromosome location, phylogeny, gene structures, gene duplication, cis-acting elements, protein-protein interaction, and expression profiles were analyzed. Twenty-five CDPK genes in the potato genome were identified based on RNA-seq data and were clustered into four groups (I-IV) based on their structural features and phylogenetic analysis. The result showed the composition of the promoter region of the *StCDPKs* gene, including light-responsive elements such as Box4, hormone-responsive elements such as ABRE, and stress-responsive elements such as MBS. Four pairs of segmental duplications were found in StCDPKs genes and the Ka/Ks ratios were below 1, indicating a purifying selection of the genes. The protein-protein interaction network revealed defense-related proteins such as; respiratory burst oxidase homologs (RBOHs) interacting with potato CDPKs. Transcript abundance was measured *via* RT-PCR between the two cultivars and their relative expression of CDPK genes was analyzed after 15, 20, and 25 days of drought. There were varied expression patterns of *StCDPK3/13/21* and *23*, between the two potato cultivars under mannitol induced-drought conditions. Correlation analysis showed that *StCDPK21/22* and *StCDPK3* may be the major differentially expressed genes involved in the regulation of malondialdehyde (MDA) and proline content in response to drought stress, opening a new research direction for genetic improvement of drought resistance in potato.

## Introduction

Plants have developed a complex signal transduction network over the years to adapt to climate change and its effects. Calcium (Ca^2+^) functions as an important secondary messenger in signal transduction during a variety of biological activities, such as growth and development (Hepler, 2005). Members of four kinase superfamilies whose activities are regulated by calcium and/or calmodulin (CaM) can sense transient fluctuations in cytoplasmic Ca^2+^ concentration. These are Calcium-dependent protein kinases (CDPKs), Ca^2+^/calmodulin-dependent protein kinases (CaMKs), calcium- and calmodulin-dependent protein kinases (CCaMKs), and CDPK-related protein kinases (CRKs) (Zuo et al., 2013; He, 2015; Wang et al., 2016; Zhang et al., 2017). Calcium-dependent protein kinases have a variable N-terminal domain that includes myristoylation or palmitoylation sites for subcellular localization (Cheng et al., 2002; Saito et al., 2018). The protein kinase catalytic domain, which has an adenosine triphosphate (ATP) binding site, is usually followed by the autoinhibitory domain, which acts as an auto inhibitor to switch CPKs between inactive and/or active states depending on the calcium concentration (Yip Delormel and Boudsocq, 2019). In addition, the calmodulin-like domain usually has four EF-hands for Ca^2+^ binding (Franz et al., 2011; Boudsocq et al., 2012) and a C-terminal domain (Hrabak et al., 2003).

Calcium-dependent protein kinases are unique sensors among Ca^2+^ sensors in that they can directly convert upstream Ca^2+^ signals directly into downstream protein phosphorylation events due to the presence of both sensory and reactive of CaM like and protein kinase domains (Poovaiah et al., 2013). Calcium ions (Ca^2+^) are released into the cytosol from internal reserves or the extracellular space under different conditions, such that different external stimuli are transduced by variable spatiotemporal differences in the frequency, amplitude, and location of Ca^2+^ waves (Kudla et al., 2018). Calcium-dependent protein kinases have been discovered throughout the plant kingdom and in some protozoa (Harper and Harmon, 2005). Calcium-dependent protein kinases are found in various subcellular locations, implying that they are involved in numerous signaling pathways (Lu and Hrabak 2002). Various stress responses and numerous environmental stimuli have been associated with increased CDPK activity/expression (Klimecka and Muszynska, 2007). Consequently, these kinases translate the information encoded in Ca^2+^ signatures into specific phosphorylation events of target proteins.

Osmotic adjustment is used by plants to respond to drought conditions; this involves the accumulation of solutes in cells in response to variations in water potential. Under various stress conditions, plants accumulate compatible solutes such as sugars, amino acids, glycerol, and mannitol, among others. As osmotic potential decreases, cells absorb water to maintain optimal turgor to support normal physiological functions (Blum, 1996; Możdżeń et al., 2021). Mannitol, an essential osmolyte, is produced in considerable amounts in many plant species (Mitoi et al., 2009), accounting in some species for almost half of all translocated photoassimilates (Loescher et al., 1992). Mannitol is a polyhydric alcohol, and beyond functioning in osmotic adjustment, mannitol also has antioxidant properties; it can scavenge hydroxyl radicals (OH^+^) (Shen, et al., 1997; Srivastava et al., 2010). Many studies of plant water relations use exposure to mannitol to experimentally induce drought stress (Soetaert et al., 1999), and mannitol simulated drought treatment is known to inhibit many physiological processes. For example, maize grown on a mannitol-supplemented medium exhibited decreased permeability of cell membranes, which was attributed to high electrolyte leakage caused by osmotic stress, and the maize plants also showed decreased chlorophyll content and inhibition of gas exchange (Możdżeń et al., 2021).

Studies of the CDPK gene family have shown functions for some of these genes in drought stress responses. For example, overexpression of *OsCDPK4/9* in rice and *AtCPK10/11* in *Arabidopsis* resulted in significantly increased drought tolerance (Campo et al., 2014; Wei et al., 2014; Zou et al., 2015). Moreover, the expression of the ginger (*Zingiber officinale*) gene *ZoCDPK1* in tobacco reduced the severity of drought stress (Vivek et al., 2017), and the overexpression of maize *ZmCPK4* in transgenic *Arabidopsis* conferred drought stress tolerance (Jiang et al., 2013). Other studies have shown that drought stress induces the transcription of the *PtrCDPK10* and *GbCDPK68* genes (Meng et al., 2020; Shi and Zhu, 2022)*.* Recently, Bi et al. (2021) reported that the expression of four potato CDPK genes (*StCDPK3/13/21/23*) is strongly induced by drought stress.

In the present study, we identified 25 *StCDPK* genes that exhibit significantly altered expression profiles upon long-term (15, 20, and 25 days) *in vitro* mannitol-induced drought stress treatment. Our study deepens the understanding of the functions of potato CDPK genes in responses to long-term mannitol-induced drought stress and lays a solid foundation for studying these genes under field conditions in drought-prone crop production regions.

## Material and Methods

### Identification and Characterization of *StCDPK* Gene Family

The protein sequences of *Arabidopsis* and rice were obtained from the NCBI database (https://www.ncbi.nlm.nih.gov/) database and a Hidden Markov Model file was created using the method BLAST (Altschul et al., 1997). The protein sequence of *StCDPK* genes was downloaded from the published potato genome database in “DM v4.04.” All candidate genes containing a kinase domain and EF-hands were identified and further verified in the Pfam (http://pfam.xfam.org/) and SMART database (http://smart.embl-heidelberg.de/) (Letunic et al., 2021). The molecular weight, theoretical pI, grand average hydropathicity, and the instability coefficient of the protein encoded by the *StCDPKs* gene were predicted using the ProtParam tool of ExPaSy (http://web.expasy.org/protparam/) (Gasteiger et al., 2005). The signal peptide of the *StCDPK* protein was analyzed using SignalP-5.0 (http://www.cbs.dtu.dk/services/SignalP/) to determine if it was a secretory protein. Interpro (http://www.ebi.ac.uk/interpro/) was used to identify *StCDPK* domains and predict the number of EF-hands present. The Palmitoylation and myristoylation sites of *StCDPK* proteins were predicted using GPS-Palm (http://csspalm.biocuckoo.org/index.php) and GPS-Lipid (http://lipid.biocuckoo.org/index.php), respectively (Ren et al., 2008; Xie et al., 2016).

### Phylogenetic Analysis of the *StCDPKs* Gene Family

A phylogenetic tree was generated by using the Neighbor-Joining method with 1,000 rapid bootstrap repeats using MEGA 7.0 (Kumar et al., 2016) using protein sequences from potato, *Arabidopsis,* and rice. The generated phylogenetic tree was visualized using Evolview v2 (http://www.evolgenius.info/evolview/).

### Chromosome Localization and Synteny Analysis

The chromosomal distribution of *StCDPKs* was identified using the MCScanX (http://chibba.pgml.uga.edu/mcscan2/) program, and the genomic positions of the *StCDPK* genes were mapped using the *S. tuberosum* database Spud DB (http://solanaceae.plantbiology.msu.edu/pgsc%20download.shtml). The map was drafted using the Mapchart program (https://www.wageningenur.nl/en.htm). The synteny blocks were used for constructing a synteny analysis map that included the genomes of potato (*S. tuberosum*), *A*. *thaliana, O. sativa*, and *S. lycopersicum.* Figures were generated using the Circos program (version 0.69) (https://circos.ca/). Gene duplication events of CDPK genes in potato were investigated. Three criteria were used in defining gene duplication: 1) the alignment length covered >80% of the longer gene, 2) the aligned region had an identity >80%, and 3) only one duplication event was counted for tightly linked genes. All of the relevant genes identified in the potato genomes were calculated using MSCanX and visualized in Circos. Based on the phylogenetic tree results, a molecular evolutionary analysis of the *StCDPK* genes was performed by calculating the nonsynonymous (Ka) to synonymous (Ks) substitution ratio of the duplicated gene pairs in *S. tuberosum* using the KaKs_Calculator in TBtools (https://github.com/CJ-Chen/TBtools) (Chen et al., 2020).

### *StCDPKs* Gene Structure and Protein Domain Analysis

To identify and draw the gene structure of the CDPKs genes, the genomic sequences were aligned with the corresponding coding sequences in the GSDS 2.0 server (http://gsds.cbi.pku.edu.cn). Proteins that share motifs within the *StCDPK* family were identified using the Multiple Expression motifs for Motif Elicitation (MEME) motif search tool (http://memesuite.org/tools/meme) (Bailey et al., 2009). They were then visualized using TBtools software.

### Analysis of Cis-Acting Elements in the Promoters of Members of the *StCDPKs* Gene Family

The 1,500 bp genomic sequence upstream of the transcription start site of the CDPKs gene family members was obtained from the potato genome database (http://solanaceae.plantbiology.msu.edu/index.shtml) and the cis-acting elements in the promoter region of *StCDPKs* gene family members were predicted by the PlantCARE online tool (http://bioinfomatics.psb.ugent.be/webtools/plantcare/html).

### Protein-Protein Network Interaction

A predicted protein-protein interaction network of the *StCDPKs* and their interacting proteins was constructed using the online program STRING V11.5 (https://string-db.org/) with the following terms; textmining, databases, co-expression, neighborhood, co-occurrence, and experimental evidence. The co-expression network was visualized using Cytoscape (Shannon et al., 2003).

### Planting Materials and Treatment Conditions

Two potato cultivars QingShu 9 (QS9) and Atlantic (Atl), known to be drought-tolerant and drought-sensitive, respectively, were used in this experiment. They were provided by the Gansu Provincial Key Laboratory of Arid Habitat Crop Science/Gansu Provincial Key Laboratory of Crop Genetic Improvement and Germplasm Innovation. Uniform plantlets from two nodal cuttings were cultured on Murashige and Skoog potato growth medium (Murashige and Skoog, 1962), which contained 5 g L^-1^ of agar, 30 g L^-1^ of sucrose, and 0.1 g L^−1^ of inositol. The pH of the medium was adjusted to 5.8 and autoclaved at 121°C at 15 1b psi for 25 min. *In vitro* propagated plants were maintained in a growth chamber at 25 ± 1°C, with a 16 h photoperiod, with active photosynthetic radiation of 45 µmol photons m^−2^s^−1^, and with a relative humidity of 55–66% for a 30-day growth period. Healthy and good-looking plantlets were then selected for stress treatment in MS medium supplemented with 150 mM mannitol for simulated drought treatment and control conditions in sterilized glass bottles (120 × 50 mm). The experiment was replicated three times, with each glass bottle containing five cuttings of potato plantlets and subjected to growth conditions 25 ± 1°C, with a 16 h photoperiod, with active photosynthetic radiation of 45 µmol photons m^−2^s^−1^, and with a relative humidity of 55–66%. Sampling was done on the 15th, 20th, and 25th days, respectively. Collected samples were immediately frozen in liquid nitrogen and stored at–80°C prior to subsequent RNA extraction.

### Determination of Phenotype and Physiological Indicators

Some physical and physiological parameters were measured after drought induction in the two potato cultivars. Plant height was measured using a meter ruler, i.e., above the root portion of the plant and total root length was measured with a root scanner (EPSON Scan 2 1200XL 2.2) and a root morphology and structure analysis device (Pornaro et al., 2017).

Oxidation stress-related traits such as malondialdehyde (MDA) content, catalase (CAT), and peroxidase (POD) activities, which have been reported as important traits related to drought tolerance in potato (Li et al., 2019), were measured after drought induction. Osmolytes such as proline was also measured after the plant was exposed to drought. Malondialdehyde (MDA content) was determined by the thiobarbituric acid (TBA) method (Heath and Packer, 1968); proline accumulation (Pro) after drought treatment was determined by the sulfosalicylic acid indandione method (Bates et al., 1973). Peroxidase activity (POD) was determined according to the Upadhyaya et al. (1985) method. In brief, POD activity was evaluated in a reaction solution containing 0.2 g of ground fresh plant tissue with 50 mM phosphate buffer, 25 mM guaiacol, and 20 mM H_2_O_2._ The amount of enzyme activity was expressed as the average change in absorbance (at a wavelength of 470 nm) per minute with readings every 1 min for a total of 3 times. Catalase activity Catalase (CAT) was measured according to the Hamurcu et al. (2013) method. In summary, an amount of 0.8 ml of stock solution was put in a 0.2 g ground sample and centrifuged for 10 min at 12,000 rpm at 4°C. The supernatant was pipetted (40 µl), and mixed with 560 µl of 0.067 M H_2_O_2_ for CAT determination. The absorbance value was measured at a wavelength of 240 nm within 1 min. All physiological analyses were repeated 3 times.

### Gene Expression Analysis

Total RNA was isolated from *in vitro* drought-stressed plants using an RNA kit (Tiangen) according to the manufacturer’s instructions. Approximately, 0.1–1 g of the plant material was collected and crushed in liquid nitrogen using a mortar and pestle. The integrity of the extracted RNA was checked on a 1% agarose gel and the concentration and purity were determined using the nucleic acid analyzer. Then, the DNA-free total RNA was used for first-strand cDNA synthesis with the kit (TOYOBO) for gene expression, and the QuantStudio 5 fluorescent real-time quantitative PCR system was used for qRT-PCR detection and analysis. The cDNA was amplified in a total reaction volume of 20 μl; i.e, SYBR Premix Ex Taq TM 10 μl, forward primer 0.8 μl, reverse primer 0.8 μl, cDNA 2 μl, ROX Reverence Dye (2X) 0.4 μl, ddH_2_O 6 μl. PCR amplification conditions were: 95°C pre-denaturation for 30 s; 95°C denaturation for 5 s, 60°C annealing for 35 s, 40 cycles; 95°C denaturation for 15 s, 58°C annealing for 60 s, and 95°C for 15 s. After the reaction, the dissolution curve was analyzed and the specificity of product amplification was checked. Actin was used as an internal reference gene and the 2^−ΔΔCt^ method (Livak and Schmittgen, 2001) was used to calculate the relative expression of *StCDPK* genes. Each gene amplification result represents data comprising three technical replications for each of three biological replicates. Supplementary Table S1 shows the primers used for gene expression analysis.

### Data Analysis

Excel 2016 version was used to sort the data, and the IBM SPSS software version 24.0 (International Business Machine Corporation, United States) was used for statistical analysis. Statistically significant differences (*p* < 0.05) are reported in the text and shown in the figures. GraphPad Prism 7 and Excel 2016 were used for data mapping and the R software was used to draw the heat map.

## Results

### Identification and Analysis of the Basic Characteristics of the Members of the *Solanum tuberosum* Calcium-Dependent Protein Kinase Family

**FIGURE 1:**
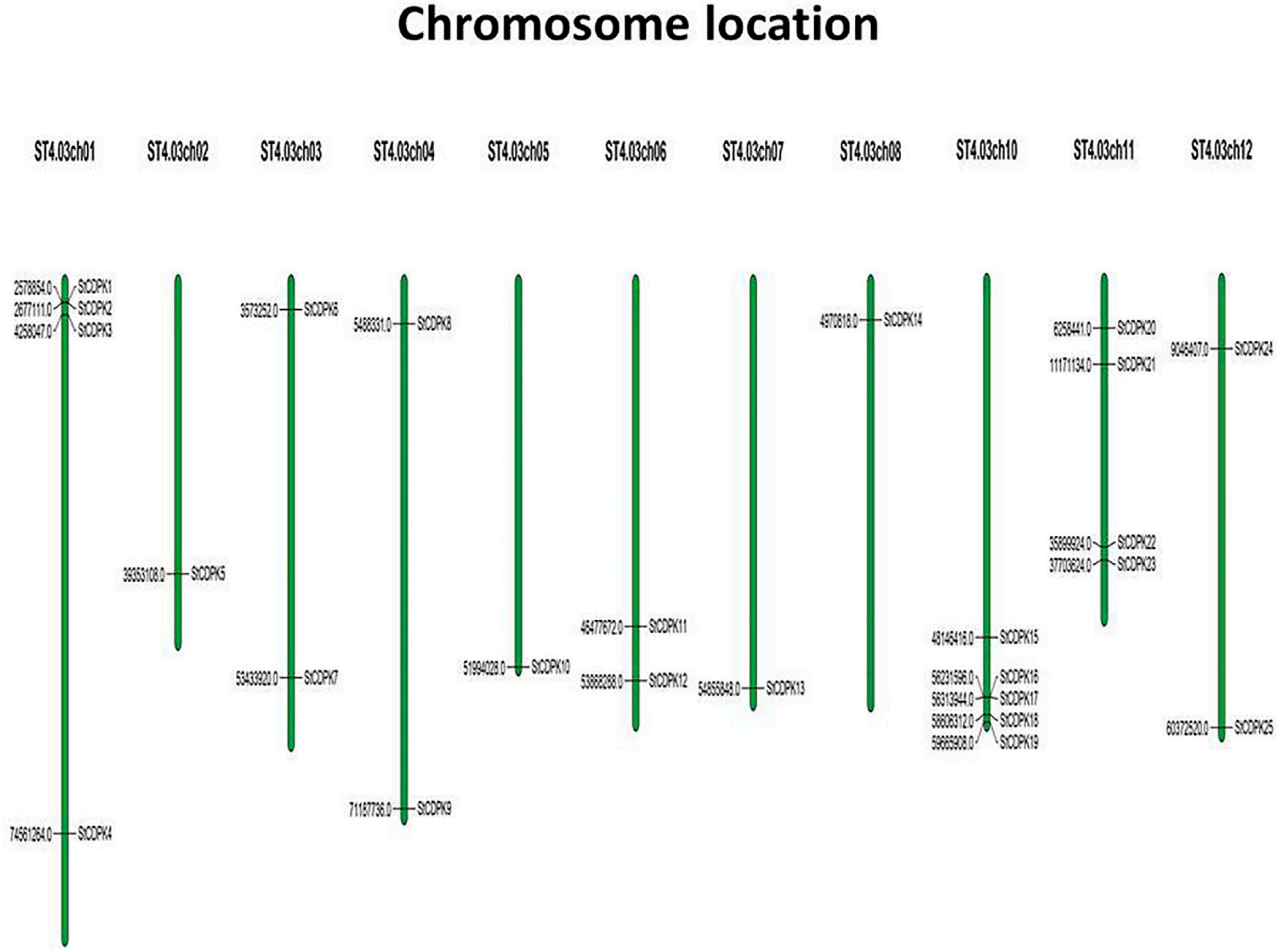
Chromosomal distribution of *StCDPK* genes in potato genome. The genes were distributed on eleven chromosomes with their numbers shown on top. The black thin lines across the chromosomes indicate the exact location of the specific gene.

A total of 25 gene loci were identified as *StCDPKs* and their coding genes were designated as *StCDPK1∼ StCDPK25* according to their location on potato chromosomes (Table 1). *In silico* analysis of the chromosomal locations of the CDPK loci indicated that the 25 CDPKs were distributed among 11 chromosomes in potato (Figure 1); only chromosome 9 lacked a CDPK locus. There were 5 *StCDPK* genes distributed on chromosome 10 (ST4.03ch10), which had the highest number of *StCDPK* loci. Two chromosomes ST4.03ch01 and ST4.03ch11 had 4 loci each; 2 loci were present on chromosomes 3, 4, 6, and 12; there was only 1 locus on chromosomes 2, 5, 7, and 8.

**TABLE 1:** The basic characteristics and Physico-chemical properties of CDPK gene family members in potato.

Subfamily type	Gene name	Gene ID	Chromosome location	CDS length (bp)	AA length (bp)	Molecular weight	pI	Instability index	GRAVY	EF-hand number	Signal peptide	Myristoylation site	Palmitoylation site
Ⅰ	StCDPK1	PGSC0003DMG400021342	1	1749	582	64,626.41	5.71	41.99	−0.408	4	No	Y	Y
	StCDPK2	PGSC0003DMG400021338	1	1797	598	67,601.16	5.42	44.41	−0.411	4	No	Y	Y
	StCDPK8	PGSC0003DMG400025435	4	1746	581	64,589.29	5.54	36.41	−0.42	4	No	Y	Y
	StCDPK10	PGSC0003DMG400023440	5	1,512	503	56,374.25	5.02	44.02	−0.28	4	No	N	Y
	StCDPK11	PGSC0003DMG400026077	6	1,506	501	56,453.55	5.69	39.53	−0.368	4	No	N	N
	StCDPK15	PGSC0003DMG400016820	10	1704	567	63,366.69	5.56	40.03	−0.374	4	No	Y	Y
	StCDPK16	PGSC0003DMG400028229	10	1824	607	68,348.03	5.58	36.32	−0.451	4	No	Y	Y
	StCDPK17	PGSC0003DMG401028133	10	1917	638	70,172.73	5.17	45.6	−0.265	4	No	Y	Y
	StCDPK19	PGSC0003DMG401007209	10	1,635	544	60,369.47	5.31	41.6	−0.411	4	No	Y	Y
	StCDPK20	PGSC0003DMG400000994	11	1737	578	64,801.71	5.35	42.82	−0.447	4	No	Y	Y
	StCDPK21	PGSC0003DMG400000890	11	1,518	505	56,997.05	5.49	37.76	−0.368	4	No	N	N
Ⅱ	StCDPK9	PGSC0003DMG400009883	4	1,425	474	53,356.88	7.89	49.51	−0.257	1	No	Y	Y
	StCDPK13	PGSC0003DMG400022318	7	1,566	521	57,862.21	6.75	29.4	−0.391	4	No	Y	Y
	StCDPK14	PGSC0003DMG400005829	8	1,554	517	57,847.71	5.79	41.08	−0.417	4	No	Y	Y
	StCDPK22	PGSC0003DMG400009451	11	1,575	524	58,971.02	5.77	40.04	−0.504	4	No	Y	Y
	StCDPK24	PGSC0003DMG400027877	12	1,530	509	56,625.8	6.07	37.42	−0.338	4	No	Y	Y
	StCDPK25	PGSC0003DMG400004646	12	1,608	535	59,662.66	5.44	42.35	−0.473	4	No	Y	Y
Ⅲ	StCDPK3	PGSC0003DMG400010704	1	1,602	532	60,004.61	6.44	37.82	−0.494	4	No	Y	Y
	StCDPK7	PGSC0003DMG400013183	3	1,617	538	60,908.99	6.43	36.81	−0.388	4	No	Y	Y
	StCDPK12	PGSC0003DMG400026908	6	1,611	536	61,050.86	5.97	30.71	−0.38	4	No	Y	Y
	StCDPK18	PGSC0003DMG400008149	10	1,575	524	59,424.7	5.95	36.01	−0.527	4	No	Y	Y
	StCDPK23	PGSC0003DMG400033335	11	1,599	532	59,737.26	6.12	38.32	−0.466	4	No	Y	Y
Ⅳ	StCDPK4	PGSC0003DMG400027527	1	1,488	495	55,392.29	5.27	39.98	−0.252	2	No	N	N
	StCDPK5	PGSC0003DMG400003564	2	1,695	564	63,596.52	9.34	39.67	−0.55	4	No	Y	Y
	StCDPK6	PGSC0003DMG400022562	3	1707	568	64,172.58	9.01	45.14	−0.63	4	No	Y	Y

AA, amino acid, pI, isoelectric point, N, no, and Y, yes.

To explore the physicochemical properties of CDPK proteins, the molecular weight (MW), isoelectric point (pI), grand hydropathy score (GRAVY), and instability index were predicted for the 25 *StCDPK* members. Predicted protein sequence analysis of the 25 *StCDPKs* revealed that the amino acid lengths ranged from 474 aa (*StCDPK9*) ∼ 638 aa (*StCDPK17*), with coding region lengths ranging from 1,425 bp (*StCDPK9*) ∼ 1917 bp (*StCDPK17*). The predicted molecular weight of the proteins ranges from 53,356.88 to 70,171.73 kDa. The differences in molecular weight can be largely attributed to the different numbers of domains. The pH at which a given molecule carries no net charge is known as the isoelectric point; this can be informative for identifying proteins with pH-dependent properties. The theoretical isoelectric points of the *StCDPKs* range from 5.02 to 9.34 (Table 1). With the exceptions of *StCDPK5, StCDPK6,* and *StCDPK9*, the predicted isoelectric points of the other *StCDPKs* proteins were below 7, a trend indicating that most of the *StCDPKs* are rich in acidic amino acids (Garcia-Moreno, 2009; Talley and Alexov, 2010).

Our analysis of the 25 *StCDPK* protein domains using the Interpro online tool (Table 1) indicated that most of the CDPK proteins were predicted to contain 4 EF-hand domains with the notable exceptions of *StCDPK4/9*, which were predicted to contain 1 and 2 EF-hand domains, respectively indicating that the arrangement of *StCDPK* protein domains is largely conserved. At the same time, the results of protein hydrophilicity analysis and instability coefficient analysis showed that the average hydrophilic GRAVY values of all *StCDPKs* were negative. The negative GRAVY values of all 25 proteins indicate a soluble or hydrophilic nature of the CDPK proteins (Kyte and Doolittle, 1982). Notably, 12 of the *StCDPKs* had protein instability coefficients predicted above 40 (Table 1), suggesting that these 12 proteins may be unstable.

We also assessed the predicted signal peptides, myristoylation sites, and palmitoylation sites for the *StCDPKs*. A signal peptide analysis showed that there were no obvious signal peptides. Prediction of potential myristoylation and palmitoylation sites indicated that 3 of the 25 CDPK proteins have no myristoylation or palmitoylation sites: whereas *StCDPK4, StCDPK10*, and *StCDPK21* were predicted to have only palmitoylation sites, the remaining 21 proteins were predicted to contain both myristoylation and palmitoylation sites (Table 1).

### Phylogenetic Tree Analysis of the Potato Calcium-Dependent Protein Kinase Gene Family

**FIGURE 2:**
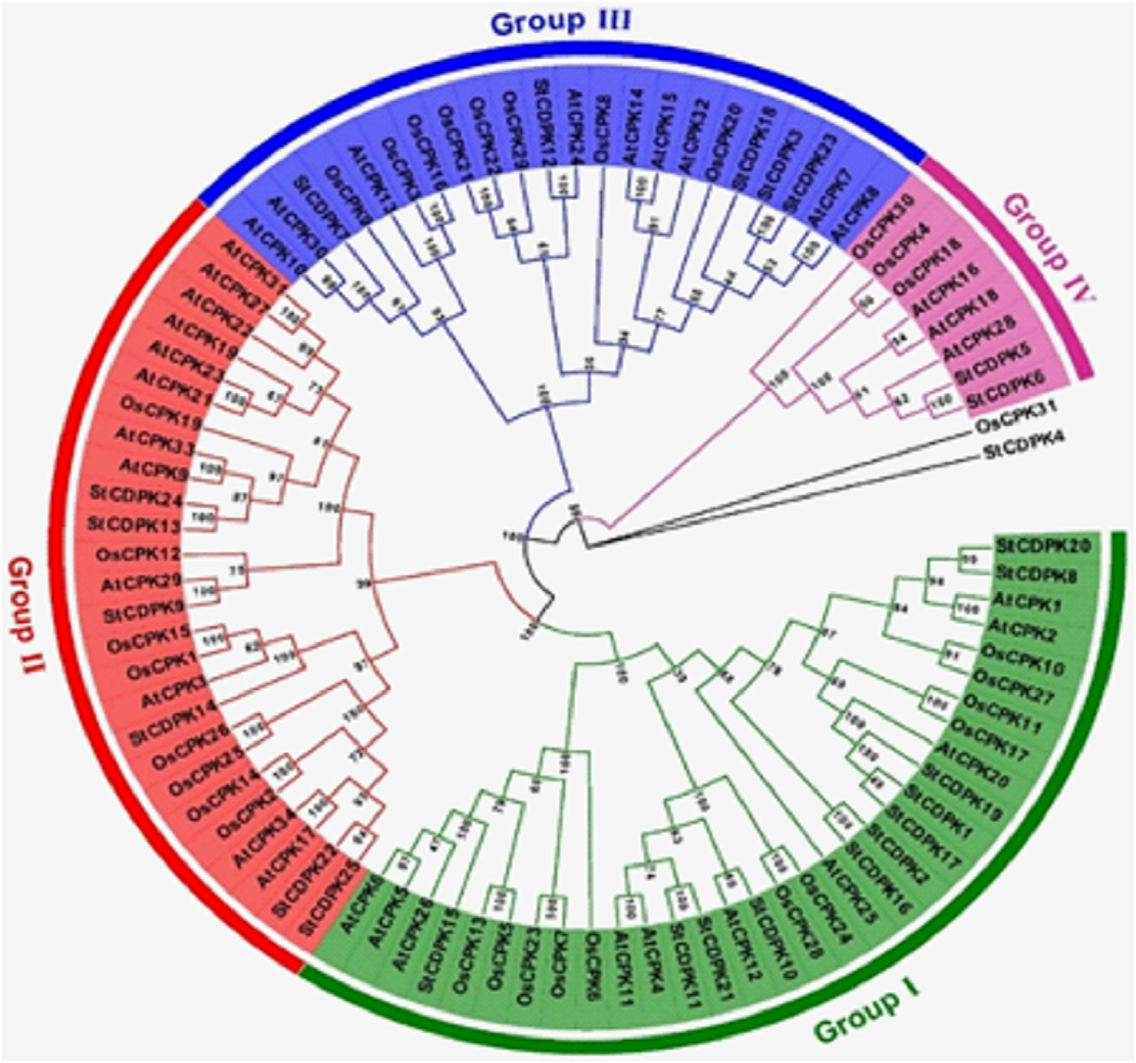
Phylogenetic tree of CDPKs gene family in potato (*Solanum tuberosum*), rice (*Oryza sativa*), and *Arabidopsis thaliana*. The tree was generated using Mega 7.0 software by the Neighbor-joining method and bootstrap analysis (1,000 replicates) expressed in percentages. Members in the same group are clustered under the same color with a chromosomal location number ranging from; *StCDPK1* to 25 in potato, *OsCPK1* to 31 in rice, and *AtCPK1* to 34 in *Arabidopsis*.

A phylogenetic tree was constructed using 90 full-length protein sequences to compare the CDPKs of potato with those of rice and *Arabidopsis* (Figure 2). Using the NCBI database search tool, 34 *Arabidopsis* and 31 rice CDPKs protein sequences were found, and a neighbor-joining tree of CDPK protein sequences from potato, *Arabidopsis*, and rice was constructed using MEGA 7.0 (Figure 2). Similar to rice and *Arabidopsis*, the potato CDPKs were clustered into four main groups (I, II, III, and IV). The largest was group I which comprised 11 *StCDPKs*, 11 *OsCPKs*, and 10 *AtCPKs.* The second group (II) comprises 6 *StCDPKs*, 8 *OsCPKs*, and 12 *AtCPKs*; the group with the fewest CDPK members was group IV, with 3 *StCDPKs*, 3 *OsCPKs*, and 3 *AtCPKs*.

### Analysis of *Solanum tuberosum* Calcium-Dependent Protein Kinases Gene Structure and Protein Domain Conservation

**FIGURE 3:**
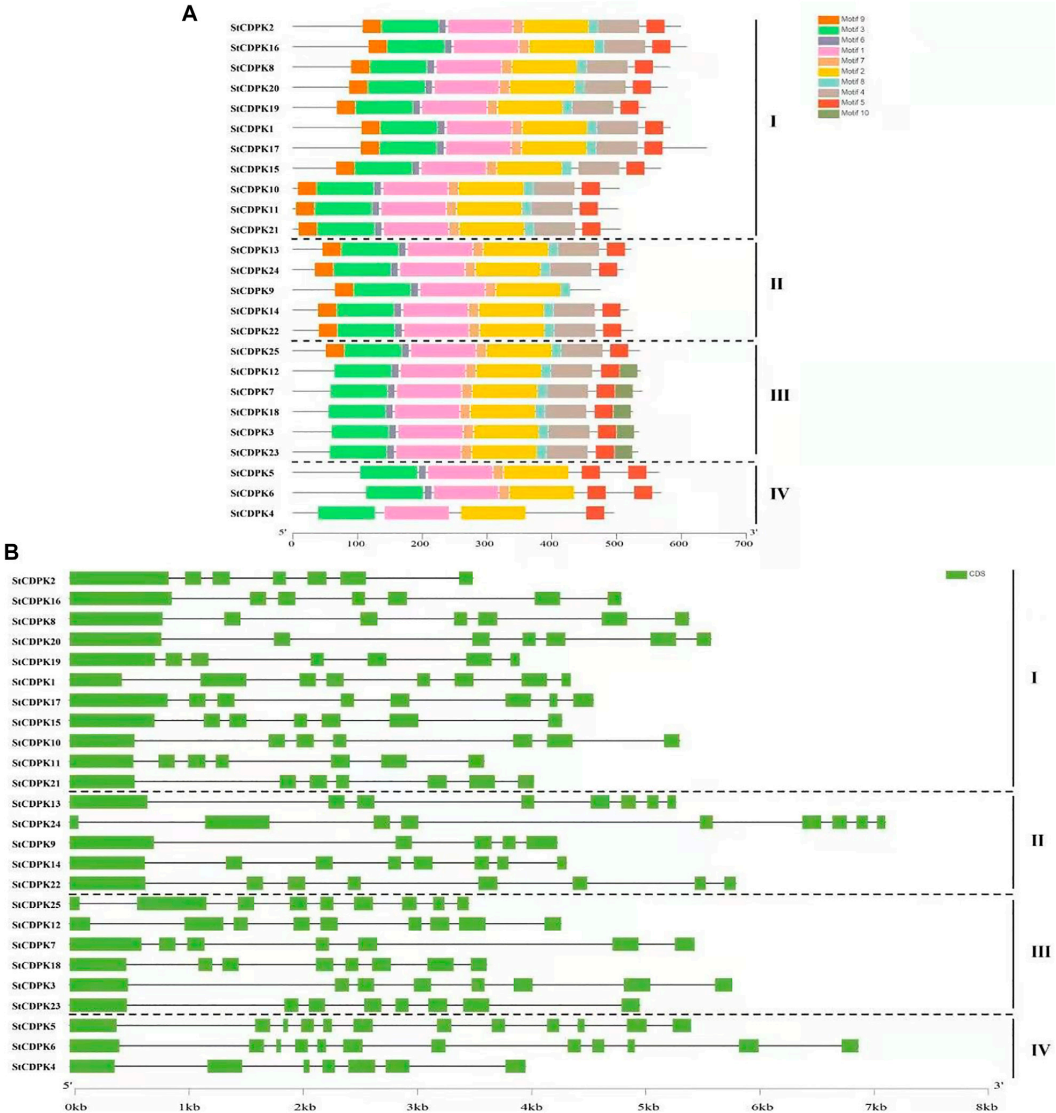
Gene structure and conserved protein motif of *StCDPKs* gene family members. **(A)** The conserved motifs of *StCDPK* proteins were discovered using the online searching tool MEME (http://memesuite.org/tools/meme). Different colors were used to indicate the conserved motifs. **(B)** Structural organization of exon/intron of the 25 potato CDPKs. Exons are represented by green boxes and black lines for introns.

Gene structure analysis of the *CDPK* genes in potato was conducted to assess gene family expansion and divergence. The number of introns varied from 5 to 12 (Figure 3B). Our analysis showed that most of the 25 *StCDPK* genes contained 7-9 introns; only the group I gene *StCDPK9* had 5 introns, while the group IV genes *StCDPK5* and *StCDPK6* each contained 12 introns. There were large differences in gene length, exon/intron structure distribution, and fragment length (Figure 3B). Using the MEME website to predict and analyze the conserved motifs of potato CDPKs, a total of 10 conserved motifs were identified (Figure 3A). Of the 10 identified motifs, motifs 1, 2, and 3 occurred in all 25 proteins, followed by motifs 5, 6, and 7, which occurred in all proteins except *StCDPK9* and *StCDPK4*; motif 10 was present in only 5 *StCDPK* proteins. Groups I and II contain similar motifs, except *StCDPK9*, which lacks motif 5; all members of group III contain motif 10 but lack motif 9. The proteins of group IV have the lowest number of motifs (Figure 3A). More than 80% of the *StCDPKs* contain motifs 1–8. Moreover, one protein *StCDPK4* contained only 4 of the 10 motifs.

### Gene Duplication, Synteny, and Ka/Ks Analysis of *StCDPK* Genes

To examine the effect of duplications on the *CDPK* gene family, we assessed putative tandem duplication and segmental duplication gene pairs using PGDD (Plant Genome Duplication Database), and visualized the results using Circos. We identified 4 pairs of paralogous segmental duplicated genes distributed on different chromosomes (Supplementary Figure S2). The identified CDPK gene pairs were *StCDPK1/StCDPK16* found on chromosomes 1 and 10, *StCDPK5/StCDPK6* located on chromosomes 2 and 3, *StCDPK20/StCDPK8* located on chromosomes 11 and 4, and *StCDPK13/StCDPK24* located on chromosomes 7 and 12. No tandem duplication was found among the potato *CDPK* genes.

To explore evolutionary relationships among the *CDPK* genes in potato, a synteny analysis that included *Arabidopsis*, rice, and tomato was performed using MCScanX. The synteny analyses help in gaining knowledge of the evolutionary and functional relationship between orthologs. This analysis found a synteny of 8 potato chromosomes with *Arabidopsis*, 11 potato chromosomes with tomato, and 1 chromosome with rice. Potato showed maximum synteny with tomato and *Arabidopsis* and least with rice because of the divergence along with the evolutionary time frame (Supplementary Figure S2). The lowest synteny found in potato and rice is due to the distant evolutionary relationship between dicots and monocots plants.

To estimate the divergence time of potato CDPKs, synonymous (Ks) and nonsynonymous (Ka) substitutions between paralogous gene pairs were calculated using the KaKs_Calculator in TBtool. Ka/Ks ratio <1, Ka/Ks = 1, and Ka/Ks > 1.00 respectively indicate purifying, neutral, and accelerated evolution with positive formation (Li, et al., 2009). In total 4 pairs of paralogous genes were found in potato CDPKs. The calculated Ka/Ks ratio of the gene pairs varied from 0.11—0.82 (Supplementary Table S2). All the paralogous *StCDPK* gene pairs had a Ka/Ks ratio <1, indicating a purifying formation (Yang et al., 2006). The estimated divergence time ranges from 20.32 to 39.32 million years ago (MYA).

### Analysis of Cis-Acting Elements in Promoters of Calcium-Dependent Protein Kinase Genes

**FIGURE 4:**
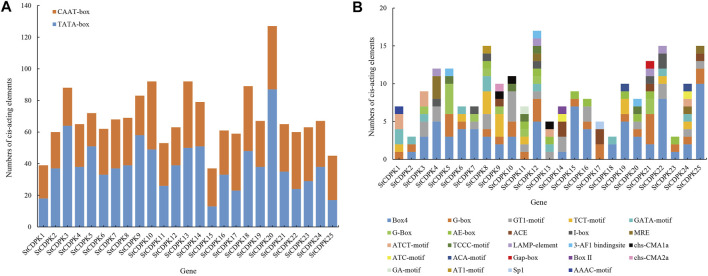
**(A)** Distribution maps of core promoter elements of the *StCDPKs* family. The core promoters were divided into two groups; CAAT-box and TATA-box indicated by brown and blue colors respectively. The PlantCARE website and database were used to identify the promoter elements of the 25 *StCDPK* genes. **(B)** Distribution map of photoresponsive functional elements in the promoters of the *StCDPKs* family. The analysis revealed 24 photo-responsive elements distributed across the identified CDPK genes. Each gene carries at least 2 photoresponsive elements at its promoter site as revealed by the PlantCARE (http://bioinfomatics.psb.ugent.be/webtools/plantcare/html) online tool.

Promoter cis-elements influence the initiation of gene transcription. We performed a bioinformatics analysis to identify possible cis-elements in the promoter sequences of *StCDPKs*. PlantCARE was used to identify putative cis-acting elements in the 1,500 bp upstream sequence of each *StCDPK* gene promoter. The results indicated that key components of the *StCDPKs* gene family include core promoter elements (Figure 4A) and plant-inducible promoter elements. In addition to nuclear promoter elements, we detected putative functional elements including light response elements, hormone response elements, stress response elements, and some growth and development-related regulatory elements.

**FIGURE 5:**
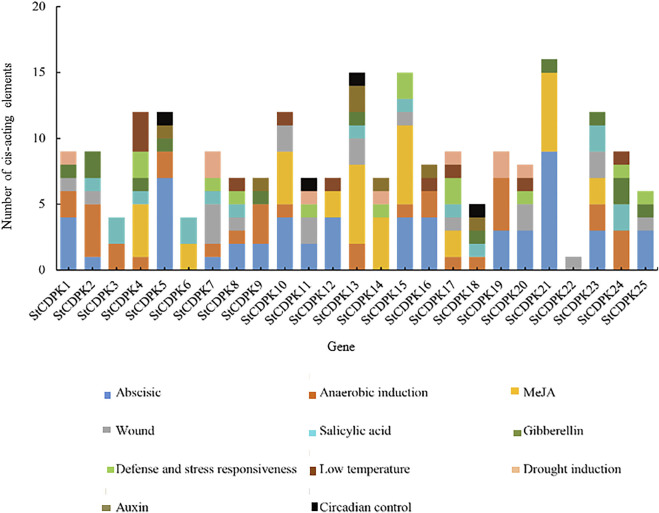
Distribution of stress-related cis-elements in the promoters of the *StCDPKs* family. The elements were partitioned into different categories; biotic responsive (defense, wound), abiotic responsive (drought induction, anaerobic induction, low temperature), and phytohormones responsive (abscisic, auxin, salicylic acid, MeJA, gibberellin). These stress-related elements are indicated by different color boxes in the chart.

Among them, the types and numbers of light-responsive elements are the largest, and these are present in the promoter regions of each *CDPK* gene, including box 4, G-box, GT1 motifs, TCT motifs, and GATA motifs (Figure 4B). Some *StCDPKs* have unique light-responsive elements, such as AAAC motifs, AT1 motifs, chs-CMA2a, GA motifs, Sp1, Box II, and Gap-box elements, which were found only in *StCDPK1, StCDPK8, StCDPK9, StCDPK11, StCDPK17, StCDPK14*, and *StCDPK21*. Interestingly, our results revealed five hormone-dependent elements in the promoters of the *StCDPKs* (Figure 5), including an abscisic acid-responsive element (ABRE), a methyl jasmonate responsive element (CGTCA-motif, TGACG-motif), a salicylic acid-responsive element (TCA-element), a gibberellin responsive element (TATC-box, GARE-motif, P-box), and an auxin-responsive element (TGA-element, TGA-box, AuxRR-core, AuxRE). Among these five types of hormone-responsive elements, 64% of the *StCDPKs* contain abscisic acid response elements, making it the most frequent hormone-responsive element; the number of methyl jasmonate response elements is second at 40%

In addition, 68% of the *StCDPK*s were predicted to contain anaerobically inducible elements (AREs): 52% of the members contained the WUN motif; 40% of the members contained defense and stress response elements (TC-rich repeats); 32% and 28% of the family members contained low-temperature response elements (LTR) and drought response elements (MBS), respectively. The *StCDPK*s with these elements appears likely to exert functions when potatoes are exposed to low temperature or drought stress (Figure 5).

### Protein-Protein Interaction Network of *StCDPK*s

**FIGURE 6:**
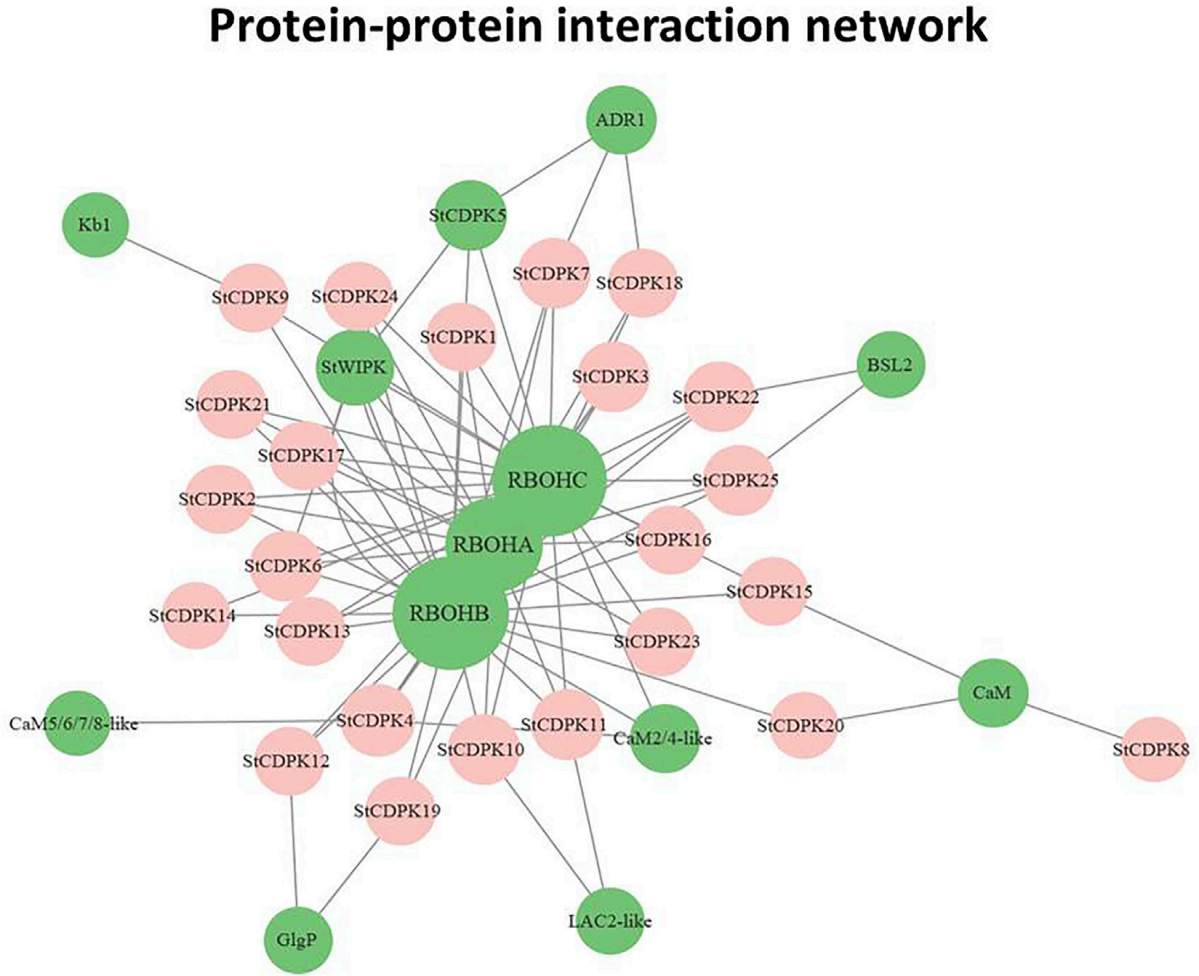
Protein-protein interaction network of *StCDPK*s. A complex network of all potato CDPKs and proteins identified and green labeled circles contains the interacting proteins.

Network interaction analysis can be an effective method for studying gene function (Zhao et al., 2018). We used the STRING 11.5 (https://string-db.org/cgi/) database to predict an interaction network for the *StCDPK* proteins (Szklarczyk et al., 2019), and Cytoscape was used to visualize the network. The network can help in connecting proteins of interest to other pathways. The network indicated that potato CDPKs apparently interact with several respiratory burst oxidase homolog proteins (RBOH), which are known to promote ROS scavenging (Foreman et al., 2003) (Figure 6). In particular, the network indicated interactions between potato CDPKs and RBOHA, RBOHB, and RBOHC, proteins with known functions in defense responses (Zhang et al., 2018) and plant development (Torres and Dangl, 2005). *StCDPK5/6* was predicted to interact with *StWIPK*, a mitogen-activated protein kinase that may be involved in the catalytic activity as well as other cellular processes (Kamiyoshihara et al., 2010). Additionally, CDPKs interact with activated disease resistance 1 (ADR1), a protein that mediates resistance against pathogens in a salicylic acid-dependent manner (Saile et al., 2021). *StCDPK12/19* was predicted to interact with glycogen phosphorylase (GlgP), an allosteric enzyme involved in carbohydrate metabolism.

### Expression Analysis of Potato Calcium-Dependent Protein Kinases in Response to Drought Stress

**FIGURE 7:**
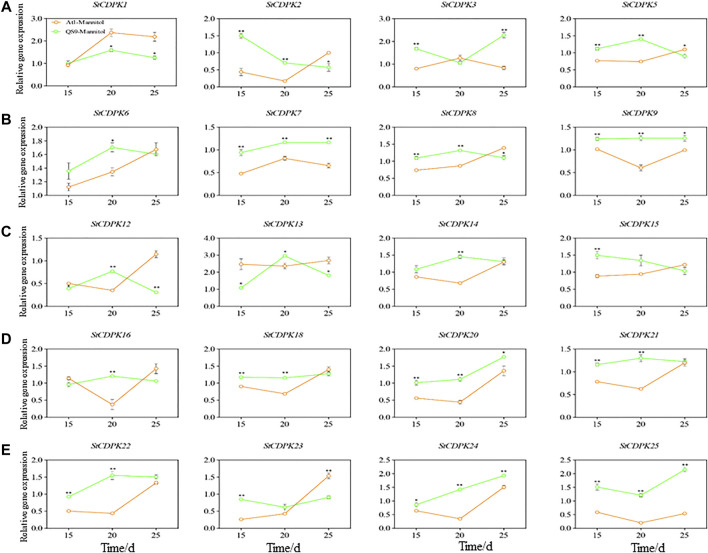
**(A-E)** Changes of *StCDPKs* relative expression in potato during different periods under long-term drought. Expression patterns of 20 CDPK genes by quantitative PCR analyses at 15, 20, and 25 days, respectively in both tolerant and drought-sensitive cultivar. The Green and orange line represents QS9 and Atlantic (Atl) cultivars, respectively. The actin gene was used as the internal control reference.

Plants are regularly threatened by abiotic and biotic stresses, and CDPKs have been described as essential factors in regulating plant tolerance to biotic and abiotic stresses (Huang et al., 2018). To gain insight into the potential functions of *StCDPKs* in abiotic stress responses, two cultivars (QS9 and Atl) with distinct drought resistance capacities (Li et al., 2019; Bi et al., 2021) were propagated in vitro with mannitol-induced drought treatment and their expression levels measured on 15, 20 and 25 days. The expression levels of the 25 *StCDPKs* were assessed by qPCR, among which 20 *StCDPK* transcripts were detected (Figures 7A-E). Moreover, 5 *StCDPK* genes (*StCDPK4, StCDPK10, StCDPK11, StCDPK17, and StCDPK19*) were not detected in either of the examined potato cultivars grown with mannitol-induced drought treatment. The expression of 20 *StCDPKs* were altered in response to stress, and the expression levels of seven of these genes increased significantly in the drought-resistant cultivar QS9 (*StCDPK7/9/20/21/22/24/25*) as compared to the drought-sensitive cultivar Atl (*p* < 0.05). Expression of the genes *StCDPK2/5/8/18/23* was significantly higher in QS9 than Atl at 15 and 20 days (*p* < 0.05) (Figures 7A-E).

### Correlation Analysis Between *StCDPK* Gene Expression and Phenotypic Changes Under Drought Stress

**FIGURE 8:**
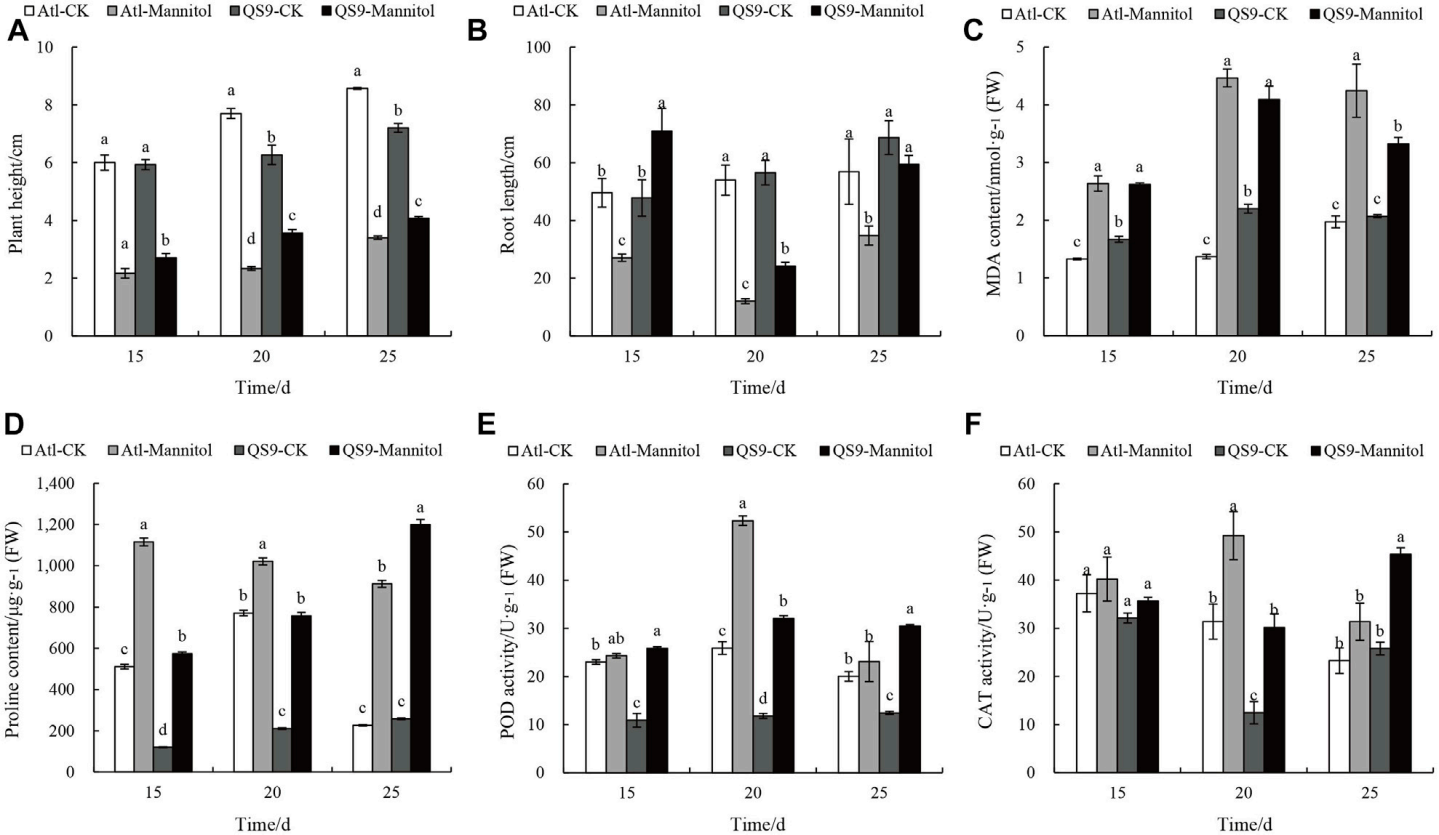
Changes in phenotypic and physiological indexes of potato test-tube plantlets in different periods under drought treatment. **(A,B)** height of plants and root length in control and mannitol-induced drought treated media measured in cm. **(C)** MDA content (nmol/g FW) of QS9 and Atl under stressed and unstressed conditions. **(D)** Proline content (μg/g FW) under drought conditions. **(E,F)** POD and CAT activities (U/g FW) in mannitol-induced drought treatment and control. Different letters show significant differences between treatments. Duncan’s method was used for significance analysis for multiple comparisons (*p* < 0.05)

We assessed changes in physiological indicators in the Atl and QS9 cultivars after 15, 20, and 25 days of drought. The plant height and total root lengths of the Atl plants were each significantly lower than those of QS9 under all the examined drought durations (*p* < 0.05) (Figures 8A,B). We also examined the levels of peroxidase (POD) and catalase (CAT) in Atl and QS9 under drought and normal conditions. In Atl and QS9, a high accumulation of these two antioxidants occurred after drought treatment. The POD activity of Atl and QS9 increased by 5.46%, 136.73%, 101.93%, and 171.41% 53.10%, and 75.00%, respectively. The POD activity in QS9 increased significantly (Figures 8E). On the 20th day of treatment, the CAT activities of Atl and QS9 were significantly higher than those of control (*p* < 0.05) (Figures 8F). The effect of cultivar x mannitol interaction on MDA activities was also significant (*p* < 0.05) in the two potato cultivars (Figure 8C). Compared to their corresponding unstressed control samples, the percentage increases in MDA content at 15, 20, and 25 days were respectively 98.21%, 56.95%, and 225.61% for the Atl plants and 85.89%, 115.09%, and 60.45% for the QS9 plants; note that the increases in MDA content in the Atl plants were significantly higher than for QS9 for all of the sampling days (*p* < 0.05). Proline is a proteogenic amino acid that is often used as a stress marker in plants where unstressed plants were reported to contained low content of proline per gram of plant tissue, whereas stressed plants contained high amount of proline (Hossain et al., 2016). We measured the proline content in the two potato cultivars after drought treatment and compared the stressed plants to their corresponding unstressed controls, the Alt plants proline increases by 118.14%, 376.64%, and 32.5% at the 15, 20, and 25-day drought stress time points, respectively, while the QS9 plants had increases of 258.91%, 302.87%, and 364.29% (Figures 8D).

**FIGURE 9:**
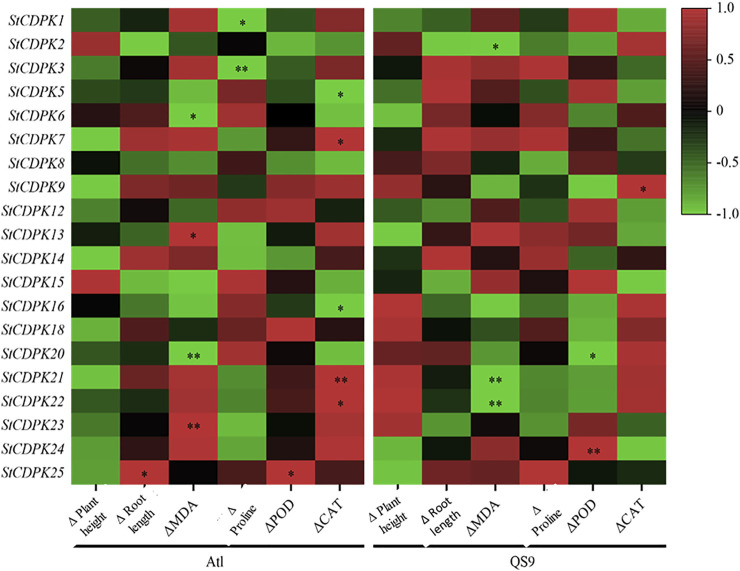
tCorrelation analysis between StCDPKs gene expression and physiological and biochemical indexes of Atl and QS9 under drought treatment. Six phenotypic and physiological parameters were examined on 15, 20, and 25 days, respectively. Red color indicates positive correlation, black color represents no correlation, and green color shows a negative correlation. * indicates significant correlation at *p* < 0.05 level, while ** indicates a highly significant correlation at *p* < 0.01 level.

We also conducted a correlation analysis for the expression of *StCDPK* genes in Atl and QS9 with the detected changes in physiological indicators under drought stress (Figure 9). We detected negative correlations between the expression levels of the *StCDPK9/18/20/21/22/23* genes in Atl with the plant height values, whereas positive correlations for plant height were detected for QS9 plants. The *StCDPK9/21/22* levels in Atl plants were positively correlated with MDA levels, whereas negative correlations were detected for these genes in the QS9 plants. In drought-stress-treated Atl plants, the expression levels of the gene pairs *StCDPK13/23* and *StCDPK6/20* were, respectively, positively and negatively correlated with the detected increases in MDA content (*p* < 0.05). Also the detected increases in MDA content (*p* < 0.05) were negatively correlated with the expression levels of *StCDPK2/21/22* in QS9 (Figure 9). Proline accumulation in QS9 correlated positively with the expression of the gene *StCDPK3/7/13/14*. There was a negative correlation between the *StCDPK1/3* expression in Atl and the detected increase in proline content (*p* < 0.05), but a positive correlation was detected between *StCDPK2* expression and the change in root length (*p* < 0.05) in Atl. POD activity in QS9 and the expression of *StCDPK24* showed a strong positive correlation (*p* < 0.01).

## Discussion

Calcium-dependent protein kinase (CDPK) is a type of serine/threonine-protein kinase found in plants and some protists. With the completion of the whole genome sequencing of several species, the CDPK gene family has been identified and cloned in a variety of plants. The genomes of *Arabidopsis*, rice, maize, wheat, tomato, melon, cucumber, and cotton are; 34, 31, 40, 20, 29, 18, 19, and 41 CDPK members, respectively (Ray et al., 2007; Li et al., 2008; Boudsocq and Sheen, 2013; Kong et al., 2013; Li et al., 2015; Xu et al., 2015; Hu et al., 2016; Zhang et al., 2017). We found 25 CDPK genes in the potato genome in our present study which is in line with (Bi et al., 2021). Phylogenetic analysis revealed four groups, with Group I being the largest group with 31 gene members from potato, rice, and *Arabidopsis,* while group IV was the smallest with 9 members (*Arabidopsis*: 3, rice: 3, potato 3). Hamel et al. (2014) observed similar grouping patterns in monocotyledons and dicotyledons.

The palmitoylation and myristoylation sites are often located at the N-terminal domains for membrane attachment (Cheng et al., 2002). Previous studies have shown that the myristoylation site is related to membrane localization, and the palmitoylation site can be used as a posttranslational signal to maintain this membrane binding (Martín and Busconi, 2001; Simeunovic et al., 2016). The results of predicting the acylation sites of the protein encoded by the *StCDPK* genes showed that 22 of the 25 *StCDPK* proteins contain both myristoylation and palmitoylation sites, tentatively suggesting that most potato CDPKs are membrane-anchored and may be involved in regulating subcellular localization. Calcium-sensing proteins have two EF-hands, each of which is a critical functional unit for protein stability and enables high-affinity calcium ion binding (Luan et al., 2002). When calcium ions bind to the EF-hands, the globular shape of CaM proteins changes, allowing CaMs to interact with their target proteins (Yamniuk and Vogel 2005). In this study, 23 *StCDPK* proteins contained 4 EF-hand domains excluding 2 proteins.

All eukaryotes evolved from a common ancestor, and during genome evolution, there might be substantial loss and gain of introns driven by selection pressure and population growth (Lynch and Conery, 2000). Structural divergences, such as the presence and position of domains, and the organization of exons/introns, can reveal evolutionary history within a gene family and are also closely related to protein function (Boudet et al., 2001; Kudla et al., 2010). The number of introns varied from 5 to 12 in potato, as in *H. brasiliensis* (Xiao et al., 2017), indicating similarities in CDPK gene structure between different species. Therefore, the presence of more introns could increase the functional diversity of CDPK genes through alternative splicing and exon shuffling (Keren et al., 2010). Similarities in the genes intron phases of the genes indicate common ancestry. During evolution, a change in the intron phase reflects a divergence in homology between genes. Long et al. (1998) found that insertion or deletion of a short DNA fragment can affect the transcript, resulting in a change in gene function. The results of gene structure and conservative protein sequence analysis showed that the gene length and exon-intron structure distribution of *StCDPK* gene family members are quite different, which may be one of the reasons for the functional differentiation among *StCDPK* gene family members.

Gene duplication is a main driving force in plant evolution, resulting in gene family expansion. Gene duplication models include segmental/whole-genome duplication (WGD), tandem duplication, proximal duplication, transposed duplication, and dispersed duplication (Paterson et al., 2010). In our analysis, we found 4 pairs of segmental duplication in *S. tuberosum* CDPKs. Zhao et al. (2021) however, discovered six pairs of segmental duplication in *M. truncatula* CDPKs. Segmental duplication gene pairs were also identified in rice (Asano et al., 2005) and poplar (Zuo et al., 2013) CDPK and CRK gene families. The Ka/Ks ratio was used to calculate the selection pressure on the duplicated gene pairs. The Ka/Ks ratio of the 4 paralogous gene pairs was less than 1in our report. This is similar to what has been observed in tomato (Hu et al., 2016), *Brassica rapa* (Wu et al., 2017), and *M. truncatula* (Zhao et al., 2021). According to the findings, these gene pairs have been subjected to strong purifying selection, which may have resulted in limited functional divergence.

Several cis-acting elements responsible for phytohormones, stress, light, growth, and development were observed in the promoter regions of *StCDPKs*, indicating the possible role of *StCDPKs* in regulating various responses to phytohormones, environmental stresses, and development. These cis-acting elements are used to predict the binding sites of transcription factors in the promoter region of genes and provide clues for the study of gene function. Analysis of the cis-acting elements in the promoter region of the *StCDPK* gene family in this study showed that the CDPK gene family contains two key promoter elements, namely the TATA box (which links transcription initiation to RNA polymerase) and the CAAT box (which regulates gene transcription efficiency), suggesting that the potato CDPK gene family can be transcribed normally. In *Arabidopsis thaliana* and *Medicago truncatula*, some *AtCPKs* and *MtCDPKs* are involved in the regulation of phytohormone and abiotic stress signaling when specific cis-acting elements were detected in the promoter regions (Li et al., 2016; Huang et al., 2018; Zhang et al., 2020; Zhao et al., 2021). Moreover, the promoter regions of different members of the potato CDPK family contain different cis-acting elements for stress response, suggesting that different members of potato CDPK may play important roles in different stresses.

Potato *CDPK* genes are likely involved in responses to biotic and abiotic stresses, particularly pathogen defense and drought, and our interaction network analysis predicted that *StCDPKs* were engaged with RBOHs. RBOHs are integral membrane proteins that produce superoxide anions that can be subsequently transformed to H_2_O_2_. RBOH proteins are known to engage in enzyme activity regulation based on their two calcium-binding EF-hand motifs and several phosphorylation sites at their N-termini. In addition to their roles in abiotic stress responses, RBOHs are known to be essential in various processes including cellular growth and hypersensitivity responses (Foreman et al., 2003). The expression of RBOHs varies depending on the tissue and developmental stage (Torres and Dangl, 2005), and various stimuli determine which isoforms are activated (Valmonte et al., 2014). *AtRBOHD* was shown to be responsible for the production of ROS in response to pathogen infection in *Arabidopsis* (Angel et al., 2015). Similarly, overexpression of the RBOHB protein in *Arabidopsis* confers resistance to soil pathogens, and RBOHB is known to function in the lateral root growth of *Phaseolus vulgaris* (Arthikala and Quinto, 2018; Hawamda et al., 2020). In strawberry, *FvRBOHA* and *FvRBOHD* are needed for the accumulation of ROS during plant response to cold stress (Zhang et al., 2018), and salt and drought stress treatments were shown to significantly enhance the expression of *VvRBOHA, VvRBOHB*, and *VvRBOHC1* in grapes. Exogenous ABA treatment significantly upregulated the expression of *VvRBOHB* (Cheng et al., 2013). RBOHA expression in *P. vulgaris* has been demonstrated to increase lateral root initiation, emergence, and development; it functions to restrict the region from which lateral roots can emerge (Arthikala and Quinto, 2018). Other predicted interacting proteins from the potato CDPK network analysis include LAC2-like proteins that function in drought tolerance in *Populus euphratica* (Niu et al., 2021).

There is growing evidence that CDPKs are involved in various physiological adaptations (Klimecka and Muszynska, 2007; Zhu et al., 2007; Xu et al., 2010). This role is evident from the expression of CDPK genes in response to various stimuli such as salt, hormones, cold, drought, heat, and wounding. To understand the potential functions of certain members of CDPKs in potato plants, their expression patterns were analyzed under different stages of mannitol-induced drought treatments. Mannitol is a polyhydric alcohol, and beyond functioning in osmotic adjustment, mannitol also has antioxidant properties; it can scavenge hydroxyl radicals (OH^+^) (Shen, et al., 1997; Srivastava et al., 2010). Mannitol-induced drought stress affects many phenotypic and physiological processes in plants (Soetaert et al., 1999; Możdżeń et al., 2021 **)**. Potato is generally considered sensitive to prolonged drought. However, the cultivar QS9 used in this study is considered tolerant to drought while the cultivar Atl cultivar is sensitive to drought stress (Li et al., 2019; Bi et al., 2021). Therefore, a better understanding of the molecular response to drought is needed to develop more tolerant cultivars.

Evaluation of the phenotypic and physiological indices of the two potato cultivars under mannitol-induced drought treatment revealed different levels of stress responses during the experimental period. The plants responded to the mannitol induced stress as shown by the results of qRT-PCR analysis, which revealed an increase in the expression levels of *StCDPK2/3/7/9/13/20/21/24/25* in cultivar QS9 after drought treatment at different periods, while, the expression levels of *StCDPK1/12/16/21/23* in cultivar Atl were also increased under drought stress. This report suggests that *StCDPK* proteins play a role in the tolerance of these cultivars to drought stress and supports the relationships among sequence, structure, and protein function. *StCDPK13* was placed in the same phylogenetic group II as *AtCPK21, AtCPK23*, and *AtCPK33*, which have been reported to be involved in drought tolerance in *Arabidopsis* (Ma and Wu, 2007; Geiger et al., 2011; Wang et al., 2019). *StCDPK3/23* which Bi et al. (2021) reported as having high expression levels in mannitol-induced drought stress was grouped in the same phylogenetic group III with *OsCPK9*, which was also reported to play a role in drought tolerance in rice (Wei et al., 2014). Thus, *StCDPK13* might be associated with physiological responses triggered by both stresses, such as regulation of stomatal movement and suppression of cell growth and photosynthesis, as has also been observed for the *FaCDPK4* gene of strawberry (Crizel et al., 2020).

The response of a plant to stress depends on the degree and duration of stress. Drought causes excessive accumulation of reactive oxygen species (ROS), leading to the induction of antioxidant enzymes such as superoxide dismutase (SOD), catalase (CAT), peroxidase (POD), malondialdehyde (MDA), and an osmolyte such as proline. The enrichment of proline content and antioxidant enzymes in the two cultivars QS9 and Atl were different. Proline content in the tolerant cultivar (QS9) increased significantly under sustained drought stress on day 25, with a corresponding decrease in MDA content on all measured days. Xu et al. (2010) reported that transgenic *Arabidopsis* lines overexpressed with *AtCPK6* had increased proline content and decreased MDA content, which is an indicator of stress tolerance. In other words, the oxidative damage associated with drought stress leads to lipid peroxidation and cell membrane dysfunction. Malondialdehyde (MDA) content was used to quantify lipid peroxidation because MDA is a typical degradation product of peroxidized polyunsaturated fatty acids in plant membranes (Ayala et al., 2014; Bhattacharjee, 2014). Simova-Stoilova et al. (2010) found an increase in catalase activity under drought and the enzyme activity appeared to be higher in the sensitive genotypes than in the tolerant ones, which is in agreement with our study where the drought-sensitive cultivar Atl showed high CAT activity after 15- and 20-day, stress exposure, while the tolerant cultivar (QS9) increased its catalase activity after 25-day drought exposure. Another study showed that the activity of CAT decreased in rice seedlings under drought (Sharma and Dubey, 2005). Moreover, POD activity increased under drought conditions in both sensitive (Atl) and tolerant (QS9) cultivars at different time intervals. Under unfavorable environmental conditions, elevated POD and CAT activities can swiftly detoxify peroxides, such as reactive oxygen species (ROS), from plants tissues, protect the cell membrane system from destruction, and delay leaves senescence thereby increasing plant resistance (Lei et al., 2006).

To better understand the relationship between gene expression, phenotype, and physiological indicators under drought, a correlation analysis was performed between the relative changes in the identified *StCDPKs*. The relative expression of the gene *StCDPK9/18/20/21/22/23* in Atl was negatively related to the relative change in plant height. Malondialdehyde (MDA) content is considered one of the most important indicators of drought stress in plants. Three genes *StCDPK9/21/22* in drought-sensitive potato (Atl) were shown to correlate positively with the relative increase in MDA content, whereas, QS9 responded negatively. With the relative expression of proline, four genes *StCDPK3/7/13/14* showed a negative correlation in Atl, while QS9 correlated positively. This indicates that these genes might be involved in regulating plant height, MDA content, and proline content through differential expression when Atl and QS9 are under drought stress to produce differences in drought resistance among cultivars. Under drought conditions, the relative expression of *StCDPK21/22* was higher in QS9 than in Atl but was negatively correlated with the relative increase in MDA content (*p* < 0.05). On the other hand, *StCDPK3* in Atl showed a negative correlation with the relative increase in proline (*p* < 0.05), suggesting that *StCDPK21/22* and *StCDPK3*, as differentially expressed genes in response to drought stress, may be involved in the regulation of MDA and proline content and could be used as candidate genes for subsequent functional research to further explore the molecular mechanism of potato response to drought.

## Conclusion

In this study, 25 copies of CDPKs were identified in the *Solanum tuberosum* genome by genome-wide structural analysis and phylogenetic characterization. Phylogenetic analysis of CDPKs from three plant species, namely, *S. tuberosum*, *Arabidopsis thaliana*, and *Oryza sativa* revealed a classification into four major groups (I-IV). Within each group, members share some common features such as protein motifs and exon-intron structures, which may be due to the fact that the members recently shared common evolutionary origins. *StCDPKs* were found to be involved in drought response and these genes showed differential expression after drought stress in the two potato cultivars studied. Gene expression profiles after drought induction detected 20 *StCDPK* transcripts. The accumulation of antioxidants in the drought-tolerant QS9 was significantly different from the sensitive cultivar Atl. The identification and systemic study of CDPK genes in *S. tuberosum* would help plant breeders to better explore the functions of CDPKs in integrating Ca^2+^ signaling pathways in *S. tuberosum* to adapt to unanticipated environmental stresses.

## Data Availability

The original contributions presented in the study are included in the article/Supplementary Material, further inquiries can be directed to the corresponding authors.

## Supplementary Material

Supplementary Figure S1 Synteny analysis of CDPK genes among *S. tuberosum* genome and *A. thaliana, O. sativa*, and *S. lycopersicum*. The *S. tuberosum* chromosome was represented by red solid blocks while *A. thaliana, O. sativa*, and *S. lycopersicum* L. were denoted by green, black, and violet colors, respectively. Collinear relationship of CDPKs between *S. tuberosum* and the other three plants was indicated by red line.

Supplementary Figure S2 Whole genome duplication distribution of *S. tuberosum* CDPK gene family. Four pairs of duplicated genes were identified and shown in red lines.

## References

[B1] Altschul S. Madden T. L. Schäffer A. A. Zhang J. Zhang Z. Miller W. (1997). Gapped BLAST and PSI-BLAST: A New Generation of Protein Database Search Programs. Nucleic Acids Res. 25, 3389–3402. 10.1093/nar/25.17.3389 9254694 PMC146917

[B3] Arthikala M.-K. Quinto C. (2018). RbohA Coordinates Lateral Root Emergence in Common Bean. Commun. Integr. Biol. 11, 1–5. 10.1080/19420889.2018.1467188 PMC6067902 30083289

[B4] Asano T. Tanaka N. Yang G. Hayashi N. Komatsu S. (2005). Genome-wide Identification of the Rice Calcium-dependent Protein Kinase and its Closely Related Kinase Gene Families: Comprehensive Analysis of the CDPKs Gene Family in Rice. Plant Cell Physiol. 46, 356–366. 10.1093/pcp/pci035 15695435

[B5] Ayala A. Muñoz M. F. Argüelles S. (2014). Lipid Peroxidation: Production, Metabolism, and Signaling Mechanisms of Malondialdehyde and 4-Hydroxy-2-Nonenal. Oxid. Med. Cell. Longev. 2014, 360438. 10.1155/2014/360438 24999379 PMC4066722

[B98] Bailey T. L. Johnson J. Grant C. E. Noble W. S. (2015). The MEME Suite. Nucl. Acids Res. 43, 39–49. 10.1093/nar/gkv416 PMC4489269 25953851

[B99] Bates L. S. Waldren R. P. Teare I. (1973). Rapid Determination of Free Proline for Water-Stress Studies. Plant Soil 39, 205–207. 10.1007/BF00018060

[B6] Bhattacharjee S. (2014). Membrane Lipid Peroxidation and its Conflict of Interest: the Two Faces of Oxidative Stress. Curr. Sci. 107, 1811–1823.

[B7] Bi Z. Wang Y. Li P. Sun C. Qin T. Bai J. (2021). Evolution and Expression Analysis of CDPK Genes under Drought Stress in Two Varieties of Potato. Biotechnol. Lett. 43, 511–521. 10.1007/s10529-020-03037-2 33131007

[B8] Blum A. (1996). “Crop Responses to Drought and the Interpretation of Adaptation,” in Drought Tolerance in Higher Plants: Genetical, Physiological and Molecular Biological Analysis (Dordrecht: Springer), 57–70. 10.1007/978-94-017-1299-6_8

[B9] Boudet N. Aubourg S. Toffano-Nioche C. Kreis M. Lecharny A. (2001). Evolution of Intron/exon Structure of DEAD Helicase Family Genes in *Arabidopsis, Caenorhabditis*, and *Drosophila* . Genome Res. 11, 2101–2114. 10.1101/gr.200801 11731501 PMC311229

[B10] Boudsocq M. Sheen J. (2013). CDPKs in Immune and Stress Signaling. Trends Plant Sci. 18, 30–40. 10.1016/j.tplants.2012.08.008 22974587 PMC3534830

[B11] Boudsocq M. Droillard M.-J. Regad L. Laurière C. (2012). Characterization of Arabidopsis Calcium-dependent Protein Kinases: Activated or Not by Calcium? Biochem. J. 447, 291–299. 10.1042/bj20112072 22827269

[B100] Campo S. Baldrich P. Messeguer J. Lalanne E. Coca M. San Segundo B. (2014). Overexpression of a Calcium-Dependent Protein Kinase Confers Salt and Drought Tolerance in Rice by Preventing Membrane Lipid Peroxidation. Plant Physiol. 165, 688–704. 10.1104/pp.113.230268 24784760 PMC4044838

[B101] Chen C. Chen H. Zhang Y. Thomas H. R. Frank M. H. He Y. Xia R. (2020). TBtools: An Integrative Toolkit Developed For Interactive Analyses of Big Biological Data. Molecular Plant 13, 1194–1202. 10.1016/j.molp.2020.06.009 32585190

[B12] Cheng S.-H. Willmann M. R. Chen H.-C. Sheen J. (2002). Calcium Signaling through Protein Kinases. The Arabidopsis Calcium-dependent Protein Kinase Gene Family. Plant Physiol. 129, 469–485. 10.1104/pp.005645 12068094 PMC1540234

[B13] Cheng C. Xu X. Gao M. Li J. Guo C. Song J. (2013). Genome-wide Analysis of Respiratory Burst Oxidase Homologs in Grape (*Vitis vinifera* L.). Int. J. Mol. Sci. 14, 24169–24186. 10.3390/ijms141224169 24351809 PMC3876103

[B14] Crizel R. L. Perin E. C. Vighi I. L. Woloski R. Seixas A. Da Silva Pinto L. (2020). Genome-wide Identification, and Characterization of the CDPK Gene Family Reveal Their Involvement in Abiotic Stress Response in Fragaria X Ananassa. Sci. Rep. 10, 11040–11117. 10.1038/s41598-020-67957-9 32632235 PMC7338424

[B16] Foreman J. Demidchik V. Bothwell J. H. F. Mylona P. Miedema H. Torres M. A. (2003). Reactive Oxygen Species Produced by NADPH Oxidase Regulate Plant Cell Growth. Nature 422, 442–446. 10.1038/nature01485 12660786

[B17] Franz S. Ehlert B. Liese A. Kurth J. Cazalé A.-C. Romeis T. (2011). Calcium-dependent Protein Kinase CPK21 Functions in Abiotic Stress Response in *Arabidopsis thaliana* . Mol. Plant 4, 83–96. 10.1093/mp/ssq064 20978086

[B18] Garcia-Moreno B. (2009). Adaptations of Proteins to Cellular and Subcellular pH. J. Biol. 8, 98–104. 10.1186/jbiol199 20017887 PMC2804283

[B19] Gasteiger E. Hoogland C. Gattiker A. Duvaud S. e. Wilkins M. R. Appel R. D. (2005). “Protein Identification and Analysis Tools on the ExPASy Server,” in The Proteomics Protocols Handbook. Totowa, NJ: Humana Press, 571–607. 10.1385/1-59259-890-0:571

[B20] Geiger D. Maierhofer T. Al-Rasheid K. A. Scherzer S. Mumm P. Liese A. (2011). Stomatal Closure by Fast Abscisic Acid Signaling is Mediated by the Guard Cell Anion Channel SLAH3 and the Receptor RCAR1. Sci. Signal. 4 (173), ra32. 10.1126/scisignal.2001346 21586729

[B21] Hamel L.-P. Sheen J. Séguin A. (2014). Ancient Signals: Comparative Genomics of Green Plant CDPKs. Trends Plant Sci. 19, 79–89. 10.1016/j.tplants.2013.10.009 24342084 PMC3932502

[B22] Hamurcu M. Sekmen A. H. Turkan İ. Gezgin S. Demiral T. Bell R. W. (2013). Induced Anti-oxidant Activity in Soybean Alleviates Oxidative Stress under Moderate Boron Toxicity. Plant Growth Regul. 70 (3), 217–226. 10.1007/s10725-013-9793-8

[B23] Harper J. F. Harmon A. (2005). Plants, Symbiosis and Parasites: A Calcium Signalling Connection. Nat. Rev. Mol. Cell Biol. 6, 555–566. 10.1038/nrm1679 16072038

[B24] Hawamda A. I. M. Zahoor A. Abbas A. Ali M. A. Bohlmann H. (2020). The *Arabidopsis* RboHB Encoded by At1g09090 Is Important for Resistance against Nematodes. Int. J. Mol. Sci. 21, 5556. 10.3390/ijms21155556 PMC7432757 32756498

[B25] He S. (2015). Genome-wide Identification and Expression Analysis of Calcium-dependent Protein Kinase and its Closely Related Kinase Genes in *Capsicum Annuum* . Front. Plant Sci. 6, 737. 10.3389/fpls.2015.00737 26442050 PMC4584942

[B26] Heath R. L. Packer L. (1968). Photoperoxidation in Isolated Chloroplasts. I. Kinetics and Stoichiometry of Fatty Acid Peroxidation. Archives Biochem. Biophys. 125 (1), 189–198. 10.1016/0003-9861(68)90654-1 5655425

[B102] Hossain M. A. Wani S. H. Bhattacharjee S. Burritt D. (2016). Drought Stress Tolerance in Plants, Vol 2. Springer, Cham. 10.1007/978-3-319-32423-4

[B27] Hepler P. K. (2005). Calcium: a Central Regulator of Plant Growth and Development. Plant Cell 17, 2142–2155. 10.1105/tpc.105.032508 16061961 PMC1182479

[B28] Hrabak E. M. Chan C. W. M. Gribskov M. Harper J. F. Choi J. H. Halford N. (2003). The *Arabidopsis* CDPK-SnRK Superfamily of Protein Kinases. Plant Physiol. 132, 666–680. 10.1104/pp.102.011999 12805596 PMC167006

[B29] Hu Z. Lv X. Xia X. Zhou J. Shi K. Yu J. (2016). Genome-wide Identification and Expression Analysis of Calcium-dependent Protein Kinase in Tomato. Front. Plant Sci. 7, 469. 10.3389/fpls.2016.00469 27092168 PMC4824780

[B30] Huang K. Peng L. Liu Y. Yao R. Liu Z. Li X. (2018). Arabidopsis Calcium-dependent Protein Kinase *AtCPK1* Plays a Positive Role in Salt/drought-Stress Response. Biochem. Biophys. Res. Commun. 498, 92–98. 10.1016/j.bbrc.2017.11.175 29196259

[B103] Jiang S. Zhang D. Wang L. Pan J. Liu Y. Kong X. (2013). A Maize Calcium-Dependent Protein Kinase Gene, Zmcpk4, Positively Regulated Abscisic Acid Signaling And Enhanced Drought Stress Tolerance In Transgenic Arabidopsis. Plant Physiol. Biochem. 71, 112–120. 10.1016/j.plaphy.2013.07.004 23911729

[B32] Kamiyoshihara Y. Iwata M. Fukaya T. Tatsuki M. Mori H. (2010). Turnover of *LeACS2*, a Wound-Inducible 1-Aminocyclopropane-1-Carboxylic Acid Synthase in Tomato, is Regulated by Phosphorylation/dephosphorylation. Plant J. 64 (1), 140–150. 10.1111/j.1365-313X.2010.otein/gzn039 20659278

[B33] Keren H. Lev-Maor G. Ast G. (2010). Alternative Splicing and Evolution: Diversification, Exon Definition and Function. Nat. Rev. Genet. 11, 345–355. 10.1038/nrg2776 20376054

[B34] Klimecka M. Muszynska G. (2007). Structure and Functions of Plant Calcium-dependent Protein Kinases. Acta Biochim. Pol. 54, 219–233. 10.18388/abp.2007_3242 17446936

[B35] Kong X. Lv W. Jiang S. Zhang D. Cai G. Pan J. (2013). Genome-wide Identification and Expression Analysis of Calcium-dependent Protein Kinase in Maize. BMC Genomics 14, 433. 10.1186/1471-2164-14-433 23815483 PMC3704972

[B36] Kudla J. Batistič O. Hashimoto K. (2010). Calcium Signals: the Lead Currency of Plant Information Processing. Plant Cell 22, 541–563. 10.1105/tpc.109.072686 20354197 PMC2861448

[B37] Kudla J. Becker D. Grill E. Hedrich R. Hippler M. Kummer U. (2018). Advances and Current Challenges in Calcium Signaling. New Phytol. 218, 414–431. 10.1111/nph.14966 29332310

[B38] Kumar S. Stecher G. Tamura K. (2016). MEGA7: Molecular Evolutionary Genetics Analysis Version 7.0 for Bigger Datasets. Mol. Biol. Evol. 33, 1870–1874. 10.1093/molbev/msw054 27004904 PMC8210823

[B39] Kyte J. Doolittle R. F. (1982). A Simple Method for Displaying the Hydropathic Character of a Protein. J. Mol. Biol. 157, 105–132. 10.1016/0022-2836(82)90515-0 7108955

[B104] Lei Y. Yin C. Li C. (2006). Differences in Some Morphological, Physiological, And Biochemical Responses To Drought Stress In Two Contrasting Populations Of Populus Przewalskii. Physiol. Plantarum 127, 182–191. 10.1111/j.1399-3054.2006.00638.x

[B40] Letunic I. Khedkar S. Bork P. (2021). SMART: Recent Updates, New Developments, and Status in 2020. Nucleic Acids Res. 49, 458–460. 10.1093/nar/gkaa937 33104802 PMC7778883

[B41] Li A.-L. Zhu Y.-F. Tan X.-M. Wang X. Wei B. Guo H.-Z. (2008). Evolutionary and Functional Study of the CDPK Gene Family in Wheat (*Triticum aestivum* L.). Plant Mol. Biol. 66, 429–443. 10.1007/s11103-007-9281-5 18185910

[B42] Li J. Zhang Z. Vang S. Yu J. Wong G. K.-S. Wang J. (2009). Correlation between Ka/Ks and Ks Is Related to Substitution Model and Evolutionary Lineage. J. Mol. Evol. 68, 414–423. 10.1007/s00239-009-9222-9 19308632

[B43] Li L.-b. Yu D.-w. Zhao F.-l. Pang C.-y. Song M.-z. Wei H.-l. (2015). Genome-wide Analysis of the Calcium-dependent Protein Kinase Gene Family in *Gossypium Raimondii* . J. Integr. Agric. 14 (1), 29–41. 10.1016/s2095-3119(14)60780-2

[B44] Li C.-L. Wang M. Wu X.-M. Chen D.-H. Lv H.-J. Shen J.-L. (2016). THI1, a Thiamine Thiazole Synthase, Interacts with Ca^2+^-dependent Protein Kinase CPK33 and Modulates the S-type Anion Channels and Stomatal Closure in Arabidopsis. Plant Physiol. 170, 1090–1104. 10.1104/pp.15.01649 26662273 PMC4734576

[B45] Li X. Ramírez D. A. Qin J. Dormatey R. Bi Z. Sun C. (2019). Water Restriction Scenarios and Their Effects on Traits in Potato with Different Degrees of Drought Tolerance. Sci. Hortic. 256, 108525. 10.1016/j.scienta.2019.05.052

[B105] Livak K. J. Schmittgen T. D. (2001). Analysis of Relative Gene Expression Data Using Real-Time Quantitative PCR and the 2^− ΔΔCT^ Method. Methods 25, 402–408. 10.1006/meth.2001.1262 11846609

[B46] Loescher W. H. Tyson R. H. Everard J. D. Redgwell R. J. Bieleski R. L. (1992). Mannitol Synthesis in Higher Plants: Evidence for the Role and Characterization of a NADPH-dependent Mannose 6-phosphate Reductase. Plant Physiol. 98, 1396–1402. 10.1104/pp.98.4.1396 16668806 PMC1080363

[B47] Long M. De Souza S. J. Rosenberg C. Gilbert W. (1998). Relationship between "Proto-Splice Sites" and Intron Phases: Evidence from Dicodon Analysis. Proc. Natl. Acad. Sci. U.S.A. 95, 219–223. 10.1073/pnas.95.1.219 9419356 PMC18181

[B48] Lu S. X. Hrabak E. M. (2002). An *Arabidopsis* Calcium-dependent Protein Kinase Is Associated with the Endoplasmic Reticulum. Plant Physiol. 128, 1008–1021. 10.1104/pp.010770 11891256 PMC152213

[B49] Luan S. Kudla J. Rodriguez-Concepcion M. Yalovsky S. Gruissem W. (2002). Calmodulins and Calcineurin B–like Proteins: Calcium Sensors for Specific Signal Response Coupling in Plants. Plant Cell 14, S389–S400. 10.1105/tpc.001115 12045290 PMC151268

[B50] Lynch M. Conery J. S. (2000). The Evolutionary Fate and Consequences of Duplicate Genes. Science 290, 1151–1155. 10.1126/science.290.5494.1151 11073452

[B51] Ma S.-Y. Wu W.-H. (2007). *AtCPK23* Functions in *Arabidopsis* Responses to Drought and Salt Stresses. Plant Mol. Biol. 65, 511–518. 10.1007/s11103-007-9187-2 17541706

[B52] Martín M. L. Busconi L. (2001). A Rice Membrane-Bound Calcium-dependent Protein Kinase Is Activated in Response to Low Temperature. Plant Physiol. 125, 1442–1449. 10.1104/pp.125.3.1442 11244123 PMC65622

[B53] Meng L. Zhang Q. Yang J. Xie G. Liu J.-H. (2020). *PtrCDPK10* of *Poncirus Trifoliata* Functions in Dehydration and Drought Tolerance by Reducing ROS Accumulation via Phosphorylating PtrAPX. Plant Sci. 291, 110320. 10.1016/j.plantsci.2019.110320 31928664

[B54] Mitoi E. Holobiuc I. Blindu R. (2009). The Effect of Mannitol on Antioxidative Enzymes *In Vitro* Long Term Cultures of *Dianthus Tenuifolius* and *Dianthus Spiculifolius* . Rom. J. Biol. Plant Biol. 54, 25–30.

[B55] Możdżeń K. Bojarski B. Rut G. Migdałek G. Repka P. Rzepka A. (2021). Effect of Drought Stress Induced by Mannitol on Physiological Parameters of Maize (*Zea mays* L.) Seedlings and Plants. J. Microbiol. Biotechnol. Food Sci. 2021, 86–91. 10.15414/jmbfs.2015.4.special2.86-91

[B56] Murashige T. Skoog F. (1962). A Revised Medium for Rapid Growth and Bio Assays with Tobacco Tissue Cultures. Physiol. Plant. 15, 473–497. 10.1111/j.1399-3054.1962.tb08052.x

[B57] Niu Z. Li G. Hu H. Lv J. Zheng Q. Liu J. (2021). A Gene that Underwent Adaptive Evolution, LAC2 (LACCASE), in *Populus Euphratica* Improves Drought Tolerance by Improving Water Transport Capacity. Hortic. Res. 8, 88. 10.1038/s41438-021-00518-x 33795664 PMC8016922

[B58] Paterson A. H. Freeling M. Tang H. Wang X. (2010). Insights from the Comparison of Plant Genome Sequences. Annu. Rev. Plant Biol. 61, 349–372. 10.1146/annurev-arplant-042809-112235 20441528

[B59] Poovaiah B. W. Du L. Wang H. Yang T. (2013). Recent Advances in Calcium/calmodulin-Mediated Signaling with an Emphasis on Plant-Microbe Interactions. Plant Physiol. 163, 531–542. 10.1104/pp.113.220780 24014576 PMC3793035

[B60] Pornaro C. Macolino S. Menegon A. Richardson M. (2017). WinRHIZO Technology for Measuring Morphological Traits of Bermudagrass Stolons. Agron. J. 109, 3007–3010. 10.2134/agronj2017.03.0187

[B61] Ray S. Agarwal P. Arora R. Kapoor S. Tyagi A. K. (2007). Expression Analysis of Calcium-dependent Protein Kinase Gene Family during Reproductive Development and Abiotic Stress Conditions in Rice (*Oryza Sativa* L. *Ssp. Indica*). Mol. Genet. Genomics 278, 493–505. 10.1007/s00438-007-0267-4 17636330

[B62] Ren J. Wen L. Gao X. Jin C. Xue Y. Yao X. (2008). CSS-Palm 2.0: An Updated Software for Palmitoylation Sites Prediction. Protein Eng. Des. Sel. 21, 639–644. 10.1093/protein/gzn039 18753194 PMC2569006

[B64] Saile S. C. Ackermann F. M. Sunil S. Keicher J. Bayless A. Bonardi V. (2021). *Arabidopsis* ADR1 Helper NLR Immune Receptors Localize and Function at the Plasma Membrane in a Phospholipid Dependent Manner. New Phytol. 232 (6), 2440–2456. 10.1111/nph.17788 34628646

[B65] Saito S. Hamamoto S. Moriya K. Matsuura A. Sato Y. Muto J. (2018). N -myristoylation and S -acylation Are Common Modifications of Ca^2+^ -regulated Arabidopsis Kinases and Are Required for Activation of the SLAC1 Anion Channel. New Phytol. 218, 1504–1521. 10.1111/nph.15053 29498046

[B66] Shannon P. Markiel A. Ozier O. Baliga N. S. Wang J. T. Ramage D. (2003). Cytoscape: A Software Environment for Integrated Models of Biomolecular Interaction Networks. Genome Res. 13 (11), 2498–2504. 10.1101/gr.1239303 14597658 PMC403769

[B67] Sharma P. Shanker Dubey R. (2005). Modulation of Nitrate Reductase Activity in Rice Seedlings under Aluminium Toxicity and Water Stress: Role of Osmolytes as Enzyme Protectant. J. Plant Physiol. 162, 854–864. 10.1016/j.jplph.2004.09.011 16146311

[B68] Shen B. Jensen R. G. Bohnert H. J. (1997). Increased Resistance to Oxidative Stress in Transgenic Plants by Targeting Mannitol Biosynthesis to Chloroplasts. Plant Physiol. 113, 1177–1183. 10.1104/pp.113.4.1177 9112772 PMC158240

[B106] Shi G. Zhu X. (2022). Genome-Wide Identification And Functional Characterization of CDPK Gene Family Reveal Their Involvement In Response To Drought Stress in *Gossypium barbadense* . Peer J. 10, e12883. 10.7717/peerj.12883 35186477 PMC8833227

[B70] Simeunovic A. Mair A. Wurzinger B. Teige M. (2016). Know where Your Clients Are: Subcellular Localization and Targets of Calcium-dependent Protein Kinases. EXBOTJ 67, 3855–3872. 10.1093/jxb/erw157 27117335

[B71] Simova-Stoilova L. Vaseva I. Grigorova B. Demirevska K. Feller U. (2010). Proteolytic Activity and Cysteine Protease Expression in Wheat Leaves under Severe Soil Drought and Recovery. Plant Physiol. Biochem. 48, 200–206. 10.1016/j.plaphy.2009.11.003 20004107

[B72] Soetaert W. Vanhooren P. T. Vandamme E. J. (1999). “The Production of Mannitol by Fermentation,” in Carbohydrate Biotechnology Protocols. Humana Press, Totowa NJ, 261–275. 10.1007/978-1-59259-261-6_21

[B73] Srivastava A. K. Ramaswamy N. K. Suprasanna P. D'souza S. F. (2010). Genome-wide Analysis of Thiourea-Modulated Salinity Stress-Responsive Transcripts in Seeds of Brassica Juncea: Identification of Signalling and Effector Components of Stress Tolerance. Ann. Bot. 106, 663–674. 10.1093/aob/mcq163 20736293 PMC2958782

[B74] Szklarczyk D. Gable A. L. Lyon D. Junge A. Wyder S. Huerta-Cepas J. (2019). STRING V11: Protein-Protein Association Networks with Increased Coverage, Supporting Functional Discovery in Genome-wide Experimental Datasets. Nucleic Acids Res. 47, D607–D613. 10.1093/nar/gky1131 30476243 PMC6323986

[B75] Talley K. Alexov E. (2010). On the pH-Optimum of Activity and Stability of Proteins. Proteins 78, 2699–2706. 10.1002/prot.22786 20589630 PMC2911520

[B76] Torres M. A. Dangl J. L. (2005). Functions of the Respiratory Burst Oxidase in Biotic Interactions, Abiotic Stress and Development. Curr. Opin. Plant Biol. 8, 397–403. 10.1016/j.pbi.2005.05.014 15939662

[B2] Torres M. A. Dangl J. L. Jones J. D. Torres M. A. (2015). Arabidopsis Gp91phox Homologues AtrbohD and AtrbohF are Required for Accumulation of Reactive Oxygen Intermediates in the Plant Defense Response. Proc. Natl. Acad. Sci. U. S. A. 99, 517–522. 10.1073/pnas.012452499 PMC117592 11756663

[B77] Upadhyaya A. Sankhla D. Davis T. D. Sankhla N. Smith B. N. (1985). Effect of Paclobutrazol on the Activities of Some Enzymes of Activated Oxygen Metabolism and Lipid Peroxidation in Senescing Soybean Leaves. J. Plant Physiol. 121 (5), 453–461. 10.1016/s0176-1617(85)80081-x

[B78] Valmonte G. R. Arthur K. Higgins C. M. Macdiarmid R. M. (2014). Calcium-dependent Protein Kinases in Plants: Evolution, Expression and Function. Plant Cell Physiol. 55, 551–569. 10.1093/pcp/pct200 24363288

[B79] Vivek P. J. Resmi M. S. Sreekumar S. Sivakumar K. C. Tuteja N. Soniya E. V. (2017). Calcium-Dependent Protein Kinase in Ginger Binds with Importin-α through its Junction Domain for Nuclear Localization, and Further Interacts with NAC Transcription Factor. Front. Plant Sci. 7, 1909. 10.3389/fpls.2016.01909 28133460 PMC5233720

[B80] Wang J.-P. Xu Y.-P. Munyampundu J.-P. Liu T.-Y. Cai X.-Z. (2016). Calcium-dependent Protein Kinase (CDPK) and CDPK-Related Kinase (CRK) Gene Families in Tomato: Genome-wide Identification and Functional Analyses in Disease Resistance. Mol. Genet. Genomics 291, 661–676. 10.1007/s00438-015-1137-0 26520101

[B81] Wang X. Lv S. Han X. Guan X. Shi X. Kang J. (2019). The Calcium-dependent Protein Kinase CPK33 Mediates Strigolactone-Induced Stomatal Closure in *Arabidopsis thaliana* . Front. Plant Sci. 10, 1630. 10.3389/fpls.2019.01630 31921270 PMC6928132

[B82] Wei S. Hu W. Deng X. Zhang Y. Liu X. Zhao X. (2014). A Rice Calcium-dependent Protein Kinase *OsCPK9* Positively Regulates Drought Stress Tolerance and Spikelet Fertility. BMC Plant Biol. 14, 133. 10.1186/1471-2229-14-133 24884869 PMC4036088

[B83] Wu P. Wang W. Duan W. Li Y. Hou X. (2017). Comprehensive Analysis of the CDPK-SnRK Superfamily Genes in Chinese Cabbage and its Evolutionary Implications in Plants. Front. Plant Sci. 8, 162. 10.3389/fpls.2017.00162 28239387 PMC5301275

[B84] Xiao X.-H. Yang M. Sui J.-L. Qi J.-Y. Fang Y.-J. Hu S.-N. (2017). The Calcium-dependent Protein Kinase (CDPK) and CDPK-Related Kinase Gene Families in Hevea Brasiliensis-Comparison with Five Other Plant Species in Structure, Evolution, and Expression. FEBS Open Bio 7, 4–24. 10.1002/2211-5463.12163 PMC5221434 28097084

[B85] Xie Y. Zheng Y. Li H. Luo X. He Z. Cao S. (2016). GPS-Lipid: A Robust Tool for the Prediction of Multiple Lipid Modification Sites. Sci. Rep. 6, 28249–9. 10.1038/srep28249 27306108 PMC4910163

[B86] Xu J. Tian Y.-S. Peng R.-H. Xiong A.-S. Zhu B. Jin X.-F. (2010). *AtCPK6*, a Functionally Redundant and Positive Regulator Involved in Salt/drought Stress Tolerance in Arabidopsis. Planta 231, 1251–1260. 10.1007/s00425-010-1122-0 20217124

[B87] Xu X. Liu M. Lu L. He M. Qu W. Xu Q. (2015). Genome-wide Analysis and Expression of the Calcium-dependent Protein Kinase Gene Family in Cucumber. Mol. Genet. Genomics 290, 1403–1414. 10.1007/s00438-015-1002-1 25687625

[B88] Yamniuk A. P. Vogel H. J. (2005). Structural Investigation into the Differential Target Enzyme Regulation Displayed by Plant Calmodulin Isoforms. Biochemistry 44, 3101–3111. 10.1021/bi047770y 15723555

[B89] Yang X. Tuskan G. A. Cheng Z.-M. (2006). Divergence of the Dof Gene Families in Poplar, *Arabidopsis*, and Rice Suggests Multiple Modes of Gene Evolution after Duplication. Plant Physiol. 142, 820–830. 10.1104/pp.106.083642 16980566 PMC1630746

[B90] Yip Delormel T. Boudsocq M. (2019). Properties and Functions of Calcium‐dependent Protein Kinases and Their Relatives in *Arabidopsis thaliana* . New Phytol. 224, 585–604. 10.1111/nph.16088 31369160

[B91] Zhang H. Wei C. Yang X. Chen H. Yang Y. Mo Y. (2017). Genome-wide Identification and Expression Analysis of Calcium-dependent Protein Kinase and its Related Kinase Gene Families in Melon (Cucumis Melo L.). PLoS One 12, e0176352. 10.1371/journal.pone.0176352 28437432 PMC5402965

[B92] Zhang Y. Li Y. He Y. Hu W. Zhang Y. Wang X. (2018). Identification of NADPH Oxidase Family Members Associated with Cold Stress in Strawberry. FEBS Open Bio 8, 593–605. 10.1002/2211-5463.12393 PMC5881550 29632812

[B93] Zhang H. Liu D. Yang B. Liu W.-Z. Mu B. Song H. (2020). Arabidopsis CPK6 Positively Regulates ABA Signaling and Drought Tolerance through Phosphorylating ABA-Responsive Element-Binding Factors. J. Exp. Bot. 71, 188–203. 10.1093/jxb/erz432 31563949

[B95] Zhao P. Liu Y. Kong W. Ji J. Cai T. Guo Z. (2021). Genome-Wide Identification and Characterization of Calcium-dependent Protein Kinase (CDPK) and CDPK-Related Kinase (CRK) Gene Families in *Medicago Truncatula* . Int. J. Mol. Sci. 22, 1044. 10.3390/ijms22031044 33494310 PMC7864493

[B107] Zhao F. Li G. Hu P. Zhao X. Li L. Wei W. (2018).Identification of Basic/Helix-Loop-Helix Transcription Factors Reveals Candidate Genes Involved In Anthocyanin Biosynthesis From The Strawberry White-Flesh Mutant. Sci. Rep. 8, 1–15. 10.1038/s41598-018-21136-z 29426907 PMC5807450

[B96] Zhu S.-Y. Yu X.-C. Wang X.-J. Zhao R. Li Y. Fan R.-C. (2007). Two Calcium-dependent Protein Kinases, CPK4 and CPK11, Regulate Abscisic Acid Signal Transduction in Arabidopsis. Plant Cell 19, 3019–3036. 10.1105/tpc.107.050666 17921317 PMC2174700

[B97] Zuo R. Hu R. Chai G. Xu M. Qi G. Kong Y. (2013). Genome-wide Identification, Classification, and Expression Analysis of CDPK and its Closely Related Gene Families in Poplar (*Populus trichocarpa*). Mol. Biol. Rep. 40, 2645–2662. 10.1007/s11033-012-2351-z 23242656

