# Supplementary material for: Identification and Expression Analysis of Calcium-Dependent Protein Kinases Gene Family in Potato Under Drought Stress
Source: Front Genet. 2022 May 24;13:874397. doi: 10.3389/fgene.2022.874397 (PMC9164159; doi:10.3389/fgene.2022.874397)
Supplement: Supplementary file 1 [file Table1.docx]

**Table S1.** Primers used in the study

| Primer name | Sequence | Tm (°C) |
| --- | --- | --- |
| StCDPK1-F | 5' CAAAACCCAAGAGCATTCATC 3' | 59 |
| StCDPK1-R | 5' AGTACTCTGAGTACCACCAAATCTATT 3' |  |
| StCDPK2-F | 5' AATGTTGTTCAGAGTAATCCGCC 3' | 59 |
| StCDPK2-R | 5' CTCATCTGTCACTATAATCATAACCTG 3' |  |
| StCDPK3-F | 5' AGAACAACAGCAAACCCAACC 3' | 60 |
| StCDPK3-R | 5' TATTTTGCCTTGTTGGCTCTTT 3' |  |
| StCDPK5-F | 5' CATTCAGAATCCAATTCCAATCC 3' | 60 |
| StCDPK5-R | 5' GGACTGGTTAGTGCTTGTACGAT 3' |  |
| StCDPK6-F | 5' AGAGCGAGATGGTAAAGGTAATAG 3' | 58 |
| StCDPK6-R | 5' CTTGGGTTGGTCTTGGATGA 3' |  |
| StCDPK7-F | 5' CATAATACGAGGAGGCAAAGTG 3' | 59 |
| StCDPK7-R | 5' GTCCATGACCCAACAACTTCC 3' |  |
| StCDPK8-F | 5' GCTAATGGGTATAGGGCAGGAA 3' | 58 |
| StCDPK8-R | 5' ACAGTAACAGGAACAGGGTGGG 3' |  |
| StCDPK9-F | 5' TCAAGATAACGATGGACAAATAGAT 3' | 59 |
| StCDPK9-R | 5' GTCTACAATTCCAAGGGCTTCTC 3' |  |
| StCDPK12-F | 5' CTTGGTGATGTTAAATTGGATGA 3' | 59 |
| StCDPK12-R | 5' CTATCAGCTTGGTTGTCACTTGC 3' |  |
| StCDPK13-F | 5' TTGGGAAGGAGTTAGGAAGAGG 3' | 60 |
| StCDPK13-R | 5' CATCTTCCACATCTATCTCTGTTCTC 3' |  |
| StCDPK14-F | 5' AAAGGTAAACCCAATAATGCTAATA 3' | 60 |
| StCDPK14-R | 5' GGCTTGGTGGAGGTCTCACAT 3' |  |
| StCDPK15-F | 5' CTCCGATGAAACTTCACCATTG 3' | 60 |
| StCDPK15-R | 5' CCAACCAGGTCTTTGAACACAT 3' |  |
| StCDPK16-F | 5' GAGAATTCTACCGGTGCTGAGT 3' | 59 |
| StCDPK16-R | 5' TCATAAGCCCCTTTAATAGTAACAATA 3' |  |
| StCDPK18-F | 5' CCAAACCCATTTTCTGTTGATTA 3' | 58.5 |
| StCDPK18-R | 5' GTCAGTAGACAAATATGTAACCCCAA 3' |  |
| StCDPK20-F | 5' GGAAACCTAATCAGTCGGAGA 3' | 58 |
| StCDPK20-R | 5' TGGAGGTGGATTACCTTGAATAC 3' |  |
| StCDPK21-F | 5' TCAAGATAAGCCACCAGAACAGA 3' | 59 |
| StCDPK21-R | 5' CTAAGTCCAGCACTAGACACCCT 3' |  |
| StCDPK22-F | 5' GAAGAAACTAGGGCAAGGTCAA 3' | 59.5 |
| StCDPK22-R | 5' CACACATCCTCATAATCTTCCTTACA 3' |  |
| StCDPK23-F | 5' ACAAGGAGATGTGTTTAAGGACATAG 3' | 59 |
| StCDPK23-R | 5' AAATCAATATGTCCACGCAAAA 3' |  |
| StCDPK24-F | 5' GCCCAGAAACTCAATTACCCTT 3' | 59.5 |
| StCDPK24-R | 5' CCTCAAATGCCTTACCCAAAA 3' |  |
| StCDPK25-F | 5' TTCTGAATAAGCTCAAAAACTTTAGTG 3' | 59 |
| StCDPK25-R | 5' CTTGATTTCATAATCTGATAATTTGTTG 3' |  |
| Actin-F | 5' AGGAGCATCCTGTCCTCCTAA 3' | 60 |
| Actin-R | 5' CACCATCACCAGAGTCCAACA 3' |  |
