# Supplementary material for: Identification and Expression Analysis of Calcium-Dependent Protein Kinases Gene Family in Potato Under Drought Stress
Source: Front Genet. 2022 May 24;13:874397. doi: 10.3389/fgene.2022.874397 (PMC9164159; doi:10.3389/fgene.2022.874397)
Supplement: Supplementary file 2 [file Table2.docx]

**Table S2**

**Supplementary Table 1.** Duplication of CDPK genes in *S. tuberosum*

| Duplicated  Gene Pairs | Ka | Ks | Ka/Ks | P-Value  (Fisher) | Divergence Time (MYA) |
| --- | --- | --- | --- | --- | --- |
| *StCDPK1*/*StCDPK16* | 0.96 | 1.18 | 0.82 | 1.15E-08 | 39.20 |
| *StCDPK5*/*StCDPK6* | 0.06 | 0.73 | 0.09 | 7.65E-90 | 24.33 |
| *StCDPK8*/*StCDPK20* | 0.07 | 0.61 | 0.11 | 6.72E-70 | 20.32 |
| *StCDPK13*/*StCDPK24* | 0.08 | 0.72 | 0.11 | 2.64E-74 | 23.98 |

Ks = synonymous substitution rate; Ka = nonsynonymous substitution rate; Ka/Ks = ratio of duplicated gene pairs; MYA = millions years ago (estimated gene divergence)
